# A key genetic factor for fucosyllactose utilization affects infant gut microbiota development

**DOI:** 10.1038/ncomms11939

**Published:** 2016-06-24

**Authors:** Takahiro Matsuki, Kana Yahagi, Hiroshi Mori, Hoshitaka Matsumoto, Taeko Hara, Saya Tajima, Eishin Ogawa, Hiroko Kodama, Kazuya Yamamoto, Takuji Yamada, Satoshi Matsumoto, Ken Kurokawa

**Affiliations:** 1 Yakult Central Institute, 5-11 Izumi, Kunitachi-shi, Tokyo 186-8650, Japan; 2 Department of Biological Information, Tokyo Institute of Technology, 2-12-1 Ookayama, Meguro-ku, Tokyo 152-8550, Japan; 3 Teikyo University School of Medicine, 2-11-1 Kaga, Itabashi-Ku, Tokyo 117-8605, Japan; 4 Earth-Life Science Institute, Tokyo Institute of Technology, 2-12-1 Ookayama, Meguro-ku, Tokyo 152-8550, Japan; 5 National Institute of Genetics, Center for Information Biology, Yata 1111, Mishima, Shizuoka 411-8540, Japan

## Abstract

Recent studies have demonstrated that gut microbiota development influences infants' health and subsequent host physiology. However, the factors shaping the development of the microbiota remain poorly understood, and the mechanisms through which these factors affect gut metabolite profiles have not been extensively investigated. Here we analyse gut microbiota development of 27 infants during the first month of life. We find three distinct clusters that transition towards Bifidobacteriaceae-dominant microbiota. We observe considerable differences in human milk oligosaccharide utilization among infant bifidobacteria. Colonization of fucosyllactose (FL)-utilizing bifidobacteria is associated with altered metabolite profiles and microbiota compositions, which have been previously shown to affect infant health. Genome analysis of infants' bifidobacteria reveals an ABC transporter as a key genetic factor for FL utilization. Thus, the ability of bifidobacteria to utilize FL and the presence of FL in breast milk may affect the development of the gut microbiota in infants, and might ultimately have therapeutic implications.

## Abstract (web summary)

The factors shaping the development of infants' gut microbiota are poorly understood. Here, the authors show that alterations in gut microbiota development in infants are associated with the presence of bifidobacteria having a gene for utilisation of human milk oligosaccharides.

## Graphical abstract

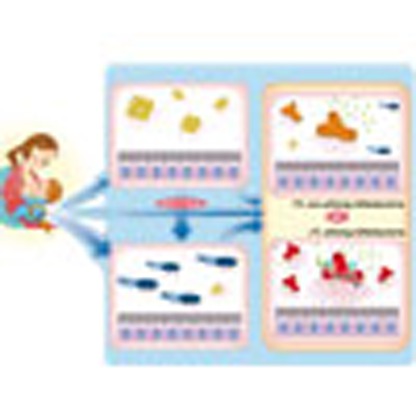

It is becoming increasingly apparent that the bacterial ecosystem in our gut has a profound influence on human health and disease. The gut microbiota contributes to immune system maturation, energy harvesting and sympathetic nervous system development. In particular, the composition and metabolite profiles of gut microbiota have been associated with pathogen resistance^1^,^2^,^3^, inflammatory responses^4^ and adiposity^5^,^6^.

Initial gut microbe colonization begins immediately after birth, and bacterial ecosystems develop within the first few days. Previous studies have reported that the composition of the infant gut microbiota differs from that of adults^7^,^8^,^9^, that substantial variation occurs between individuals^6^,^10^,^11^ and that bifidobacteria predominate in most infants^11^,^12^,^13^. Recent studies also demonstrated that environmental factors including the mode of delivery and feeding affect the gut microbiota assemblage and that the process is not random^6^,^13^,^14^. Furthermore, it has been indicated that the gut microbiota development during infancy can have long-lasting effects on the individual's future health^15^,^16^,^17^,^18^. However, little is known about their pattern of progression, factors that drive the assembly of infant gut microbiota and how these factors affect metabolite profiles.

Here we investigated gut microbiota compositions and metabolic profiles for 217 stool samples obtained from 27 infants during their first month of life (202 samples from 12 infants were analysed longitudinally and 15 samples from 15 infants were studied in follow-up). The dynamics and equilibria of the developing microbiota were investigated, and their associations with metabolites were evaluated. We subsequently analysed phenotypes and genotypes of isolated bifidobacteria, and found a key genetic factor affecting infant gut microbiota composition and metabolite profile.

## Results

### Early development of gut microbiota

To investigate the dynamics of gut microbiota immediately after birth, we analysed the sequences of the V1–V2 region of the 16S rRNA genes obtained from 12 infants born by normal delivery (Supplementary Table 1) using the 454 GS Junior platform. We obtained stool samples every day during the first week after birth and every other day thereafter until 1 month of age (∼17 stool samples per infant; 202 samples in total). A total of 588,293 pyrosequencing reads (average 2,912±1,397 reads per sample; Supplementary Table 2 and Supplementary Fig. 1) were analysed using an open-source Quantitative Insights Into Microbial Ecology (QIIME) software pipeline^19^ (Supplementary Table 3).

**Figure 1:**
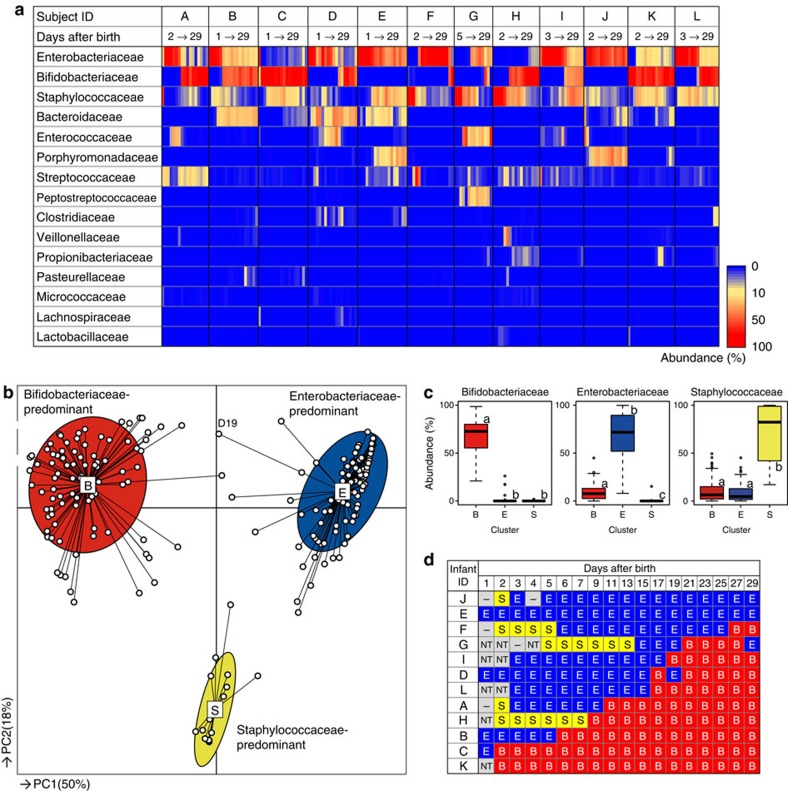
Infant gut microbiota community profiles during the first month of life. (**a**) Microbiota profiles in stool samples from 12 subjects (*n*=202; ∼17 sampling days per subject), temporally ordered from left to right. Each row represents taxonomic groups at the family level. The top 15 families are displayed and sorted according to relative abundance. Abundances are represented using the colour scale. (**b**) Characteristics of infant gut microbiota, illustrated by PCoA and PAM clustering analyses. Data from individuals (points) were clustered, and the centres of gravity (rectangles) were computed for each class. The coloured ellipses encompass 67% of the samples in each cluster. (**c**) Box plots showing the relative abundances of the main contributors to each cluster. Different letters (a–c) above the boxes indicate significant differences between clusters (*P*<0.05, Mann–Whitney *U*-test with Bonferroni's correction). (**d**) Temporal shift from Staphylococcaceae- or Enterobacteriaceae-dominant microbiota to Bifidobacteriaceae-dominant microbiota. S, Staphylococcaceae-dominated (yellow); E, Enterobacteriaceae-dominated (blue); B, Bifidobacteriaceae-dominated (red); NT, not tested; —, sample not provided.

Figure 1a shows an age-dependent, gut microbiota composition heatmap for each subject at the bacterial family level. The analysis demonstrated that there are major variations in both the composition and dynamic progression among individuals. Overall, the composition of the infant's microbiota was relatively simple, being composed of only a few dominant bacterial families. The displacement of predominant bacteria occurred within only a few days. We observed an increased average abundance of Bifidobacteriaceae, α-diversities and total bacterial cell counts, as well as decreased average abundances of Enterobacteriaceae and Staphylococcaceae (Supplementary Fig. 2).

Characteristics of the taxonomic composition observed among the samples were clearly distinguished by principal coordinate analysis (PCoA) and partitioning around medoids (PAM)^20^ on the basis of bacterial family composition data (Fig. 1b and Supplementary Data 1). Values of the Calinski-Harabasz (CH) index with PAM clustering suggest that the infant microbiota could be divided into three clusters (Supplementary Fig. 3), which were characterized by the predominance of Bifidobacteriaceae, Enterobacteriaceae or Staphylococcaceae (Fig. 1c). We subsequently observed sequential transitions occurring from Staphylococcaceae- to Enterobacteriaceae- and/or Enterobacteriaceae- to Bifidobacteriaceae-dominated microbiota, with considerable individual variation in the day of the transition (Fig. 1d). Transitions in the opposite direction were rarely observed. The results suggest that the best-adapted bacterial family in the infant gut may be Bifidobacteriaceae, followed by Enterobacteriaceae and Staphylococcaceae.

### Microbiota in 1-month-old infants

**Figure 2:**
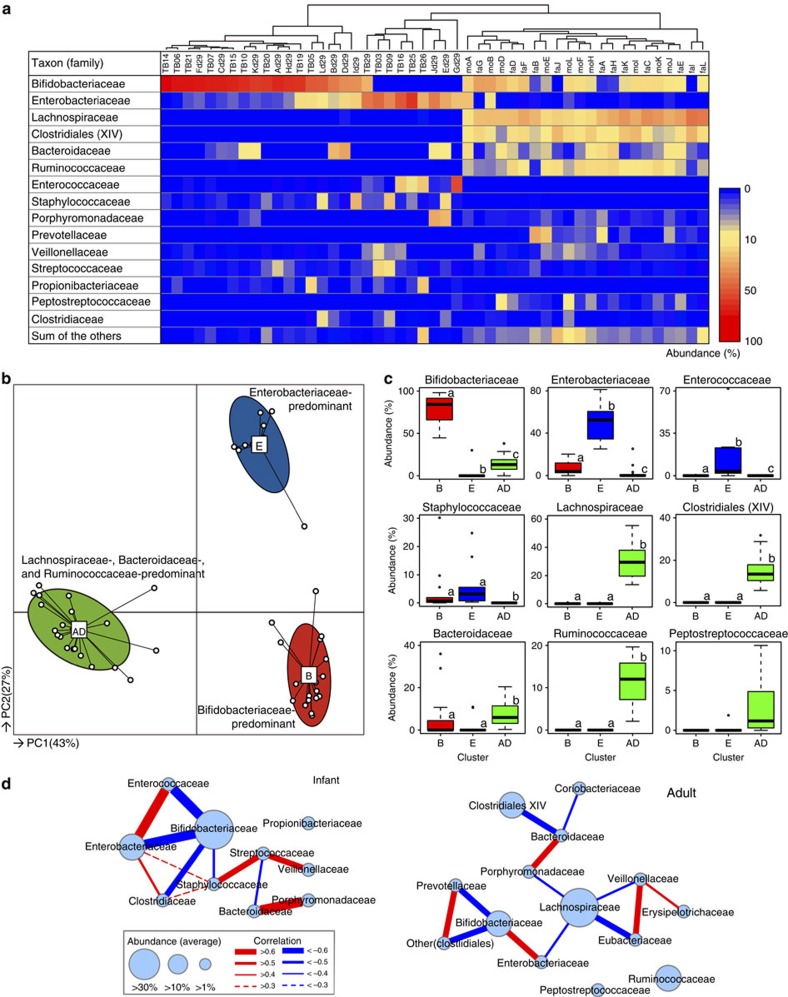
Gut microbiota community profiles of 27 1-month-old infants and 22 adults. (**a**) Bacterial families representing more than 1% (on average) of the microbiota in infants or adults are shown on the colour scale. Samples were hierarchically clustered by measuring Euclidean distances with complete-linkage clustering, as shown in the upper tree. (**b**) Characteristics of infant and adult gut microbiota, as illustrated by PCoA and PAM clustering analyses. Cluster B, Bifidobacteriaceae-predominant; cluster E, Enterobacteriaceae**-predominant; cluster AD, adult-type microbiota. (**c**) Abundances of the main contributors to each cluster. Different letters (a–c) indicate significant differences between clusters (*P*<0.05, Mann–Whitney *U*-test with Bonferroni's correction). Differences in other bacterial families are shown in Supplementary Fig. 7). (**d**) Network diagram showing co-occurrence relationships among the main contributors in infants and adults. Node sizes indicate the abundances of each bacterial family, and the widths of the edges reflect the calculated Spearman's rank correlation coefficient.

Subsequently, we performed 16S rRNA gene-library analysis using additional faecal samples; in addition to the faecal samples from 12 infants (subjects A–L, shown in Fig. 1 on day 29), we obtained faecal samples from their parents (*n*=22) and another 15 breast-fed infants at approximately 1 month after birth (Supplementary Tables 4 and 5). A total of 147,010 high-quality reads (average 3,058±1,232 reads per sample; Supplementary Data 2) were analysed, and the resulting family composition heatmap is shown in Fig. 2a.

The results of PCoA and PAM clustering based on bacterial family compositions suggest that the microbiota of 1-month-old infants stratified into two clusters that were distinct from the adult cluster (Fig. 2b and Supplementary Fig. 4). The majority of infants (*n*=18, designated as cluster B) were characterized as having a significantly high abundance of Bifidobacteriaceae, and the minor cluster (*n*=9, designated as cluster E) had a significantly high abundance of facultative anaerobes such as Enterobacteriaceae, Enterococcaceae and Staphylococcaceae (Fig. 2c and Supplementary Figs 5 and 6). The 22 adult samples formed a single cluster (designated cluster AD), showing significantly higher abundances of Lachnospiraceae, ‘Clostridiales incertae sedis XIV', Bacteroidaceae, Ruminococcaceae and Peptostreptococcaceae, as well as higher α-diversity compared with the two infant microbiota clusters (Fig. 2c and Supplementary Figs 5–7).

The correlation coefficients observed between bacterial family abundances in infant and adult microbiota are illustrated in network diagrams (Fig. 2d and Supplementary Figs 8 and 9). Analysis of the infant stool samples indicated that the abundance of predominant Bifidobacteriaceae negatively correlated with those of Enterobacteriaceae, Enterococcaceae, Clostridiaceae and Staphylococcaceae. Furthermore, the network diagram for adults indicated that bacterial family compositions and their associations were different from those of infants.

### Bacterial lineages and gut environments

**Figure 3:**
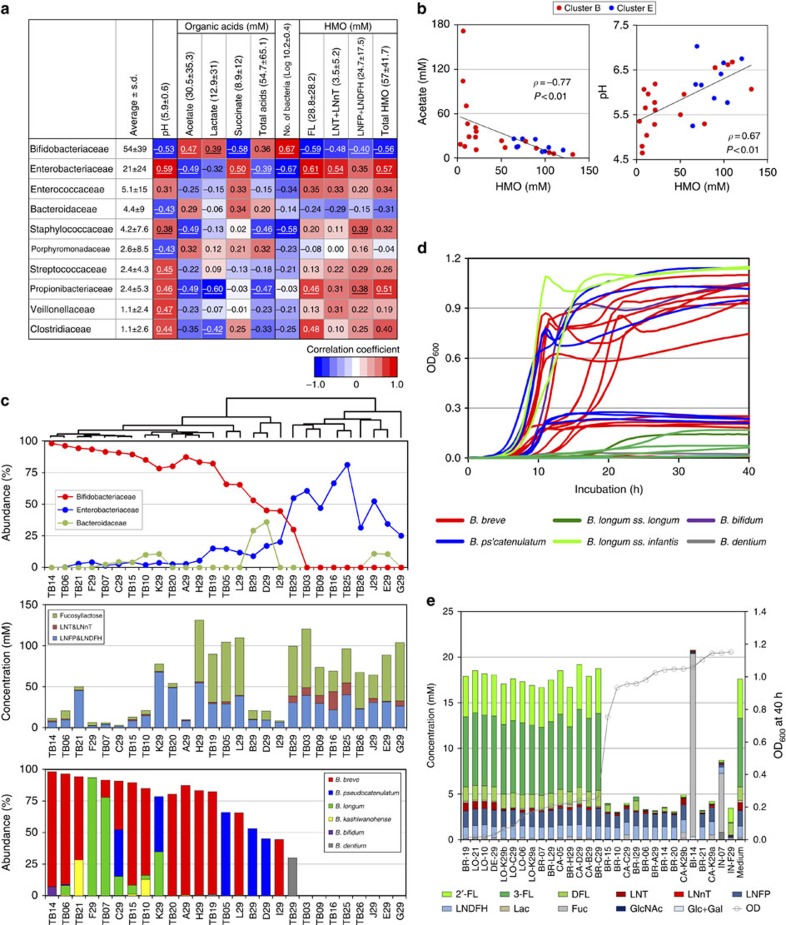
Relationships between bacterial family abundances and gut environments. (**a**) Spearman's rank correlation coefficients between bacterial family abundances and gut environmental factors such as pH, organic acid concentrations and faecal oligosaccharide concentrations are shown in numerical and colour-scale formats. (**b**) Spearman's rank correlations of oligosaccharide concentrations with pH values and acetate concentrations. (**c**) Relationships between bacterial abundances, faecal oligosaccharide concentrations and the relative abundances of bifidobacterial species. The upper tree shows hierarchical clustering on the basis of the bacterial family compositions. (**d**) Growth curves of 29 bifidobacterial strains in medium containing HMOs (see Supplementary Fig. 14 for more details). (**e**) Glycoprofiles of bacterial supernatants after 40 h of cultivation. Samples are ordered based on their OD_600_ values after 40 h of cultivation.

To understand how gut microbiota affect the host's physiology, it is important to understand the entire gut ecosystem, not only in terms of microbiota compositions but also in terms of the metabolites produced by the bacteria. Therefore, we investigated the pH and organic acid concentrations of each infant's stool (Supplementary Data 3) and assessed the correlation between these parameters and bacterial family abundances (Fig. 3a and Supplementary Fig. 10). We found that increased Bifidobacteriaceae abundance positively correlated with organic acid concentrations and total bacterial counts but negatively correlated with pH. In contrast, the abundance of facultative anaerobes such as Enterobacteriaceae and Staphylococcaceae correlated with decreased organic acid concentrations, decreased total bacterial counts and increased pH (Fig. 3a), as well as decreased Bifidobacteriaceae abundance (Fig. 2d).

Previous studies have reported that bifidobacteria produce acetate and lactate as their metabolites^1^,^21^, and that some of these strains (for example, *Bifidobacterium longum* ss. *infantis* ATCC 15697) can efficiently utilize some human milk oligosaccharide (HMO) components^22^,^23^,^24^,^25^. Therefore, we hypothesized that bifidobacteria consume the remaining oligosaccharides in the infant gut, causing elevated acetate and lactate concentrations and decreased pH. Furthermore, we examined the concentrations of major residual HMO components in infant stools by high-performance liquid chromatography (HPLC; Supplementary Data 3 and Supplementary Figs 11 and 12)^26^. As expected, HMO consumption in the gut was associated with increased abundances of Bifidobacteriaceae (Fig. 3a), increased organic acid concentrations and decreased pH values (Fig. 3b). However, some infants showed high faecal oligosaccharide concentrations despite the presence of Bifidobacteriaceae (Fig. 3c). Therefore, we examined the species composition of bifidobacteria in these infants (Fig. 3c). We also analysed the oligosaccharide profiles of breast milk provided by their mothers, and found that three infants (subjects I29, TB16 and TB19) received breast milk from non-secretor mothers (lacking 2'-fucosyllactose (2'-FL) and 2', 3-difucosyllactose (DFL), Supplementary Table 6). However, we found it difficult to explain these discordant results.

These observations prompted us to isolate bifidobacteria from the infant faeces to assess their ability to utilize HMOs *in vitro*. The 29 isolated strains were cultured in medium containing HMOs as the carbon source and their growth was monitored (Fig. 3d and Supplementary Figs 13 and 14). Interestingly, 14 of the 29 strains exhibited remarkable growth in HMO medium (saturating OD_600_>0.7), but 15 strains did not (saturating OD_600_<0.3). Culture supernatants were collected to investigate the remaining oligosaccharides. Although most bifidobacterial strains utilized lacto-*N*-tetraose (LNT), there were considerable differences in the utilization of fucosyllactose (sum of 2′-FL, 3-fucosyllactose and DFL), which is the main component of HMOs^27^,^28^ (Fig. 3e and Supplementary Fig. 15). These results indicated that efficient FL utilization is not a universal property of infant bifidobacteria and is instead strain-dependent.

### Bifidobacterial genomes and FL utilization

To gain insight into how the strains showed differences in FL utilization, we determined the draft genomes of all 29 strains (Table 1 and Supplementary Table 7) and subsequently performed OrthoMCL clustering analysis using bi-directional BLAST alignments (Supplementary Fig. 16a). Initially, we investigated the presence of fucosidase genes (glycoside hydrolase family classifications: GH95 and GH29) and confirmed that all strains exhibiting robust growth in an HMO-containing medium possessed at least one fucosidase gene (Table 1 and Supplementary Fig. 16b). However, we found that 6 out of 15 strains showing limited growth in the HMO-containing medium possessed a fucosidase gene. We analysed the phylogeny and subcellular localization of the fucosidase genes. We found that most fucosidases, except for that of *B. bifidum* BI-14, are intracellular enzymes, with only minor sequence differences occurring between FL-utilizing and non-FL-utilizing strains (Supplementary Fig. 17).

**Figure 4:**
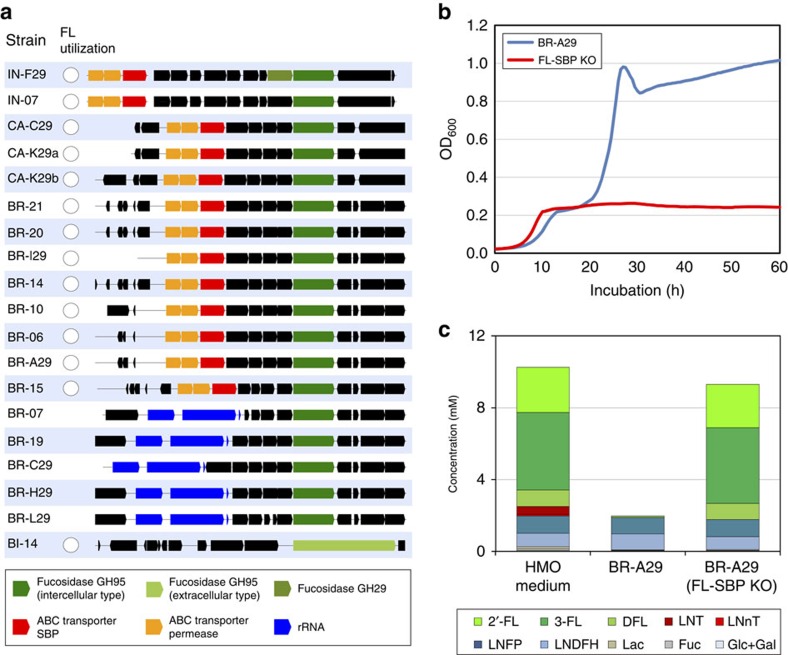
Identification of bifidobacterial genes responsible for HMO utilization. (**a**) Syntenic relationships of putative FL-utilization gene clusters identified among the bifidobacteria. The genes and their orientations are depicted with arrows. Strains lacking fucosidase genes (10 strains among 29) are not represented. (**b**) Growth curves of the *B. breve* BR-A29 strain and the corresponding FL-SBP gene knockout strain in medium containing HMOs. (**c**) Glycoprofiles of BR-A29 and FL-SBP gene-knockout strains after 40 h of cultivation.

Subsequently, we searched for other genes responsible for FL utilization and discovered that the presence of a homologous group (HG_2571) corresponded with the FL-utilization phenotype in all strains, except for *B. bifidum* BI-14 (Table 1 and Supplementary Fig. 16c). BLAST searches against the KEGG database indicated that the homologous sequences were highly similar to substrate-binding protein (SBP), which participates in the multiple-sugar ABC transporter system (KEGG entry K02027; Supplementary Fig. 16c). Furthermore, we found that two permease genes of the multiple-sugar ABC transporter system (K02025 and K02026) and the fucosidase gene (GH95) were adjacent to the SBP gene in most strains (Fig. 4a). On the basis of these findings, we hypothesized that the ABC transporter mediates FL transportation into bacterial cells. This hypothesis could explain the absence of the transporter in the *B. bifidum* BI-14 strain, which utilizes FL because that strain expresses extracellular membrane-bound fucosidases (Supplementary Fig. 17).

To determine whether the putative ABC transporter SBP for FL (denoted FL-SBP) mediates FL utilization, the gene was knocked out in *B. breve* BR-A29 using homologous recombination (Supplementary Fig. 18). After confirming that the *FL-SBP* gene was knocked out, growth of the knockout strain was investigated in the HMO medium. In contrast with the original BR-A29 strain, the FL-SBP gene-knockout strain showed limited growth in the HMO medium (Fig. 4b). Furthermore, we confirmed that FL was not utilized by the FL-SBP gene-knockout strain (Fig. 4c), demonstrating that the FL-SBP is responsible for FL utilization.

### HMO-utilizing bifidobacteria affect gut ecosystems

**Figure 5:**
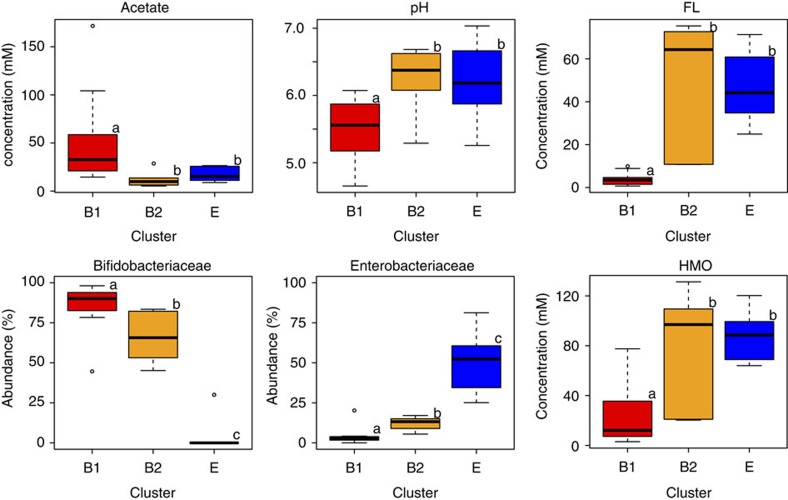
Impact of FL-utilizing bifidobacteria on gut microbial ecosystems. Box plots showing differences among Bifidobacteria-dominant microbiota, with or without FL-utilizing bifidobacteria (clusters B1 and B2, respectively) and Enterobacteriaceae-dominant microbiota (cluster E). Different letters (a–c) above the boxes indicate significant differences between clusters (*P*<0.05, Mann–Whitney *U*-test with Bonferroni's correction).

Having identified the differences in FL utilization among bifidobacteria, we subdivided the Bifidobacteriaceae-dominated microbiota (cluster B) into those colonized by FL-utilizing bifidobacteria (designated cluster B1; *n*=11) and those dominated with non-FL-utilizing bifidobacteria (cluster B2; *n*=7; Supplementary Fig. 19). Furthermore, we compared the faecal organic acids, pH, HMO and microbiota compositions of these two subgroups with those of Enterobacteriaceae-dominated microbiota (cluster E; *n*=9). Compared with clusters B2 and E, cluster B1 showed significantly higher acetate concentrations and lower pH and residual oligosaccharide concentrations (*P*<0.05, Mann–Whitney *U*-test with Bonferroni's correction; Fig. 5 and Supplementary Fig. 20). In addition, cluster B1 had significantly higher Bifidobacteriaceae and lower Enterobacteriaceae abundances. In contrast, there were no significant differences in faecal acetate concentrations, pH or oligosaccharide concentrations between clusters B2 and E.

## Discussion

Gut microbiota development in healthy, full-term infants has been investigated using culture-based enumeration^29^, molecular analysis with 16S rRNA gene-targeting primers or probes^10^,^11^,^12^,^30^, or, more recently, using metagenomic approaches^8^,^9^,^13^,^31^. These intensive observational studies have demonstrated that the modes of delivery^10^,^13^, feeding^10^,^13^,^29^ and use of antibiotics^12^ can influence the development of the infants' microbiota. In addition, recent investigations have suggested that maternal HMO secretion type^31^, environmental exposures^10^,^14^ and bacterial transmission and propagation^30^ can also alter the assemblage of infants' microbiota. However, the overall understanding of gut microbiota development is still limited because of the small number of subjects studied^9^, the low frequency of sample analysis^8^,^13^,^31^ and/or the limitations in the enumeration methods used^10^,^11^,^12^. In addition, metabolite profiles, bacterial strain isolation and related phenotype and genotype investigations were not performed in these studies. Thus, the presence of infant microbiota equilibria, key bacterial lineages and genetic factors regulating gut metabolite profiles and the molecular mechanisms that influence the gut microbiota in early life remain poorly understood.

**Figure 6:**
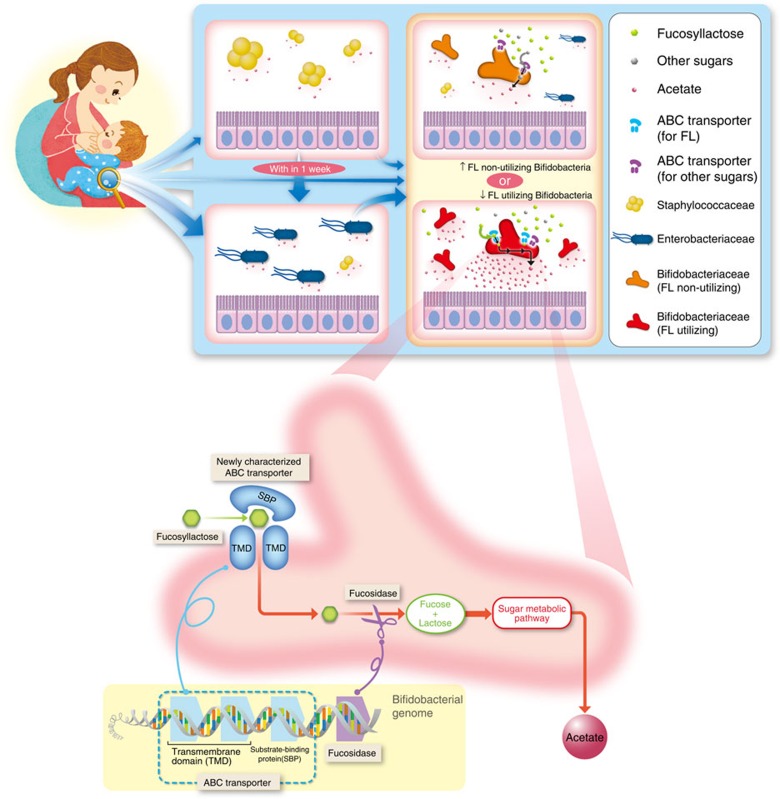
Infant microbiota development and molecular mechanisms of FL utilization by bifidobacteria. Infant gut microbiota showed 3 distinct clusters, which underwent directional transition to Bifidobacteriaceae-dominant microbiota, and displayed individual variations in the pace of progression. Isolated bifidobacterial strain showed differences in FL utilization. Colonization of FL-utilizing bifidobacteria are associated with altered gut acetate concentrations, pH, and Enterobacteriaceae and Bifidobacteriaceae abundances in the cohort, which have been previously shown to affect infant health. We subsequently identified a SBP of the multiple-sugar ABC transporter system is a key genetic factor of FL utilization.

Among the considerable number of metabolic pathways, we demonstrated the importance of the FL-utilization pathway, which is associated with high concentrations of acetate in the gut and alters the microbiota composition (Fig. 6). To our knowledge, this is the first study suggesting that a single bacterial gene may affect the gut metabolic profile and microbiota composition in a human cohort. Previous metagenomics and metabolomics analyses in infants have suggested that gut microbial communities consistently serve the same major functions, regardless of variations in their compositions^18^. In contrast, we focused on the utilization of HMOs, the most abundant carbohydrates in the infant gut, and found that the important gut microbial metabolic activity was associated with a specific group of infant gut microbes in this study.

Genomic analysis of bifidobacteria and subsequent experiments with target gene knockout strain identified an ABC transporter that plays an essential role in FL utilization. Previous studies have demonstrated that some bifidobacteria can efficiently utilize HMO components, such as LNT^22^,^23^. Differences in FL utilization have also been observed between different species and strains^32^,^33^. However, the mechanism and importance of FL transport into bacterial cells have not been demonstrated.

Recently, Lewis *et al*.^31^ investigated the relationship between maternal secretor status and the development of infant microbiota, and reported that infants fed by non-secretor mothers are delayed in the establishment of microbiota dominated by bifidobacteria. However, the FL-utilizing properties of bifidobacteria and the molecular mechanisms of FL utilization characterized in this study were not taken account. In this study, we found that 3 out of 27 infants received breast milk from non-secretor mothers, and one infant (subject I, Supplementary Fig. 19) harboured FL-utilizing bifidobacteria. The infant exhibited considerably lower Bifidobacteriaceae abundance, higher Enterobacteriaceae abundance, lower acetate concentrations and higher pH (45%, 20%, 16.2 mM and 6.2, respectively; Supplementary Data 2 and 3) compared with the other cluster B1 infants (Fig. 5). These results support our notion that the presence of FL in breast milk and colonization of FL-utilizing bifidobacteria induce altered gut microbiota composition and metabolite profiles, consistent with the observation by Lewis *et al*.^31^ Larger infant cohorts are needed to identify the relationship between maternal secretor status and colonization of FL-utilizing and non-utilizing bifidobacteria in order to draw more robust conclusion.

A recent study reported that the ‘commensal colonization factors' encoding a unique class of polysaccharide utilization genes play important roles in stable colonization of *Bacteroides*, one of the most predominant genera in the human adult microbiota^34^. In the current study, we identified the FL transporter as a key stable colonization factor of gut microbes in infants. More importantly, the FL transporter was shown to cause changes in the gut microbiome and the production of metabolites, which may confer benefits to infants. Thus, these data indicate that the FL transporter is a symbiotic colonization factor of infant bifidobacteria, which is conceptually distinct from the commensal colonization factor of *Bacteroides* colonizing the adult gut.

Changes in metabolite profiles and microbiota compositions caused by FL-utilizing bifidobacteria have been suggested to have a variety of beneficial effects on the host^1^,^4^,^35^,^36^,^37^,^38^. It has been demonstrated that the acetate produced by bifidobacteria protects against *Escherichia coli* O157 infection by improving the gut barrier^1^ or inhibiting Shiga-toxin production^35^ in animal models. Other studies have reported that the short-chain fatty acids produced by gut microbes are associated with host energy balance^5^,^36^, inflammation resistance^4^ and sympathetic nervous system development^37^. In addition, correlations between decreased Enterobacteriaceae abundance and lower susceptibility to infection have been demonstrated in animal models^2^ and human cohorts^3^. Thus, the ABC transporter that is present in HMO-utilizing bifidobacteria may benefit the host by facilitating acetate production and limiting the abundance of Enterobacteriaceae.

Given the importance of infant gut microbiota for the long-term health of the individual, the FL-utilizing properties of bifidobacteria and related molecular mechanisms may ultimately have implications in therapeutic approaches or preventive medicine. Our findings suggest that the presence of FL in breast milk and its utilization by bifidobacteria help fostering the mutually beneficial relationship between humans and the main constituents of the infant gut microbiota. Since the ABC transporter is present in only a subset of bifidobacteria, future studies should investigate the effects of FL-utilizing bifidobacteria on the health of individuals using human interventions or animal models. The phenotypic and genotypic properties of bifidobacteria should be considered during the development of probiotics targeting the gut microbiota of infants.

## Methods

### Subject recruitment and sample collection

The study was approved by the ethical committees of Yakult Central Institute (subjects A to L; Supplementary Table 1) and Teikyo University (subjects TB–XX; Supplementary Table 4). Written informed consent was obtained from the parents before enrolment. No infants received antibiotics or probiotics before or during the study period. Infant stool samples were collected by the parents, ∼17 times each from 12 infants (subjects A to L) and once from 15 infants (Supplementary Table 4). Additional stool samples were collected from most parents of infants A to L (Supplementary Table 5). The samples were frozen at −20 °C, transferred to the laboratory and stored at −80 °C. In total, 239 samples were collected for gut microbiota analysis, including 217 stool samples from infants and 22 stool samples from parents. Parents were instructed to record changes in diet, medications, hospitalizations, birth weight and gestation age at the time of delivery (Supplementary Tables 1 and 4). HMOs were purified from breast milk provided by nine healthy volunteers (32.6±3.9 years of age) at approximately 1 year following delivery.

### DNA extraction

DNA was extracted as described in ref. 39. Briefly, 20 mg of each faecal sample (equivalent to 100 μl of fivefold homogenate) was suspended in 500 μl of extraction buffer (100 mM Tris-HCl, pH 9.0, 40 mM EDTA and 1% SDS; final concentrations) in a 2-ml screw-cap tube. Then, 300 mg of 0.1-mm-diameter glass beads and 500 μl of TE buffer-saturated phenol were added to the suspension. Microbial cells were lysed by mechanical disruption using a FastPrep FP 120 (BIO 101, Vista, CA, USA) at a power level of 5.0 for 30 s. The mixtures were centrifuged at 4,500*g* for 5 min, and the upper layers were subjected to phenol/chloroform/isoamyl alcohol extraction, followed by isopropanol and ethanol precipitation. Finally, the dried DNA samples were suspended in 1 ml TE buffer and stored at −35 °C until they were pyrosequenced.

### 16S rRNA gene amplification and pyrosequencing

The V1–V2 regions of 16S rRNA gene were selected for pyrosequencing analysis because that region offers enough sequence variation for species-level discrimination of bifidobacteria, which are reported as the main constituent of infant gut microbiota. The 16S rRNA genes of each sample were amplified using a forward 63Fm-TAG-linker A primer (5′- *CCATCTCATCCCTGCGTGTCTCCGAC*- TCAG-NNNNNNNNNN-GCYTAAYACATGCAAGTMGA -3′) and a reverse 338R-linker B primer (5′- *CCTATCCCCTGTGTGCCTTGGCAGTCTCAG*-GCTGCCWCCCGTAGG-WGT -3′). The italicized sequence represents 454 Life Sciences linkers A and B, respectively; TCAG in forward primer was inserted as a key sequence as recommended by the manufacturer; NNNNNNNNNN in forward primers represents the unique 10-base barcode for tagging each PCR product; and the underlined sequence is a broadly conserved sequence in bacterial 16S rRNA genes. We used universal primer 63F (ref. 40) with modification since bifidobacteria have a two-base-pair mismatch with respect to the universal primer 27F (ref. 41). The taxonomic coverage of the 63Fm and 338Rm primer sequences was evaluated with the Probe Match programme in the Ribosomal Database Project (RDP; Release 11, Update 2)^42^. A total of 1,354,916 16S rRNA gene sequences in the RDP were retrieved using the following parameters: strain=both, source=both, size⩾1,200, quality=good. The sequences retrieved were used as reference sequences for the Probe Match programme to evaluate the taxonomic coverage of the primer pair. The taxonomic coverage of the primer pair for each phylum was calculated as the percentage of genera for which more than half of the member sequences in each genus were found to match the candidate sequence with no more than one mismatch^43^. The PCR mixture (50 μl total volume) contained 1 × SYBR Premix Ex Taq (Takara Bio, Osaka, Japan), 100 nM of each primer and 1 μl of template DNA. The thermocycling conditions used were as follows: 95 °C for 5 min, followed by 25–40 cycles of 95 °C for 30 s, 55 °C for 30 s and 72 °C for 1 min. Amplification was performed using an ABI PRISM 7500 Real-Time PCR System (Applied Biosystems, Framingham, MA, USA), and thermal cycling was stopped before the amplification curves reached a plateau to avoid introducing biases to the microbiota composition and generation of chimera. Amplicons were purified with the AMPure XP Kit (Beckman Coulter Genomics GmbH, Bernried, Germany) and quantified using the Quant-iT PicoGreen dsDNA Kit (Invitrogen, Leek, the Netherlands). Equal amounts of the amplicons were pooled and sequenced using a Roche 454 GS Junior pyrosequencer with a GS FLX Titanium emPCR Kit (Lib-L) according to the manufacturer's protocols (Roche Applied Science, Bavaria, Germany).

### 16S rRNA gene sequence-based microbiota analysis

The sequences generated from the 454 GS Junior platform were analysed using the open-source software package QIIME^19^. The resultant 16S rRNA gene sequences were assigned to operational taxonomic units (OTUs) using the USEARCH algorithm^44^, with a 97% identity threshold. Simultaneously, UCHIME^45^ was employed to remove potential chimeric sequences from a representative set of OTUs, using the reference mode (against ‘Gold' database^46^). The taxonomy of each representative OTU sequence was assigned using the RDP-naive Bayesian Classifier with a minimum bootstrap threshold of 50% (ref. 47). A single representative from each OTU was aligned using the MUSCLE alignment tool^48^, and a phylogenetic tree was constructed using FastTree^49^. α-diversities (the number of OTU observed, Shannon index and phylogenetic diversity) were estimated for 1,000 randomly selected sequences to account for differences in sampling effort between the samples. Detailed QIIME commands and options are summarized in Supplementary Table 3.

PCoA and between-class analysis were performed as described in ref. 20. Data generated by QIIME at taxonomic level 5 (family) were used to calculate the Jensen–Shannon distances between samples. The PAM clustering algorithm^50^ was applied to cluster the profiles. Estimation of the number of clusters in infant gut microbiota by PAM clustering according to the method in ref. 20 with modifications. We examined the number of clusters exhibiting the highest CH index^51^ in the randomly subsampled data set (see Supplementary Figs 3 and 4 for more details). This trial was repeated 1,000 times. A cluster number most frequently exhibiting the highest CH index in the 1,000 trials was defined as the optimal number of microbiota cluster.

### Total bacterial counts

Approximately 1 g of faecal samples were diluted fivefold with PBS, homogenized, and a portion of the homogenates (100 μl) was fixed with 300 μl of 4% paraformaldehyde at 4 °C for 16 h. The paraformaldehyde-treated samples were smeared on a MAS-coated slide glass (Matsunami, Osaka, Japan) and stained with Vectashield Mounting Medium with 4,6-diamidino-2-phenylindole (Vector Laboratories, Burlingame, CA, USA). The fluorescent cells were counted in an automated format using a Leica MM AF microscope (Leica, Wetzlar, Germany) and Image-Pro Plus Image Analysis Software (version 5.1; Media-Cybernetics, Silver Spring, MD, USA). Bacterial counts were expressed as the mean values of 10 fields for each sample.

### pH values and organic acid concentrations

The pH values of the faecal samples were measured directly with a handheld pH meter (model IQ150) equipped with a PH17SS electrode (IQ Scientific Instruments, San Diego, CA, USA). To determine the concentration of organic acids, 180μl of faecal homogenates (fivefold, prepared as described above) were mixed with 20 μl 10% HClO_4_ and incubated at 4 °C overnight. Subsequently, the samples were centrifuged for 5 min at 14,000*g* and the supernatants were filtered through Centricut W-MO membrane filters (0.45 μm; Kurabo, Tokyo, Japan). The concentrations of organic acids were determined with a HPLC system equipped with 432 electroconductivity detectors (Waters, Milford, MA, USA) and a Rspak KC-811 column (Showa Denko KK, Tokyo, Japan)^52^.

### Statistics

Statistical analyses were performed using the R software, version 3.1.0 (http://www.r-project.org/) and Excel 2013 (Microsoft). Differences in bacterial abundance, α-diversities and other host metadata between the clusters were evaluated by the Mann–Whitney *U*-test, with Bonferroni's correction. Differences between clusters were represented with box plots. The middle bar of each plot indicates the median, while the top and bottom of each box indicate the third and first quartiles, respectively. Whiskers denote the lowest and highest values within 1.5 times the interquartile range from the first and third quartiles, respectively. Circles denote outliers beyond the whiskers. Correlations between host metadata, microbiota (bacterial family abundances, α-diversities and total bacterial counts) and gut environmental factors (pH, organic acid and oligosaccharide concentrations) were analysed by calculating Spearman's rank correlation coefficients. To construct the network diagram, Spearman's correlations were computed between the bacterial families representing more than 1% (on average) of the microbiota (Supplementary Figs 8 and 9). Only relationships having an absolute Spearman's correlation above 0.3 with a *P*-value less than 0.05 were selected. The yEd Graph Editor (https://www.yworks.com/products/yed) was then used to construct the network figures.

### Preparation of HMOs

Breast milk samples (∼1 l total) were combined, mixed with 4 volumes of chloroform:methanol (2:1) and stirred vigorously for 1 min. After centrifugation at 9,000*g*, the upper layer was collected, evaporated and dissolved in H_2_O. Two volumes of ethanol were added to it and incubated overnight at 4 °C, and the precipitated lactose was removed by passaging samples through filter paper. The ethanol was removed by rotary evaporation, after which the residue was dissolved in H_2_O and applied to an XK column (5 cm in diameter and 100 cm in length; GE Healthcare, Buckinghamshire, UK). Elution was performed overnight using water as the mobile phase, with a flow rate of 1.5 ml min^−1^. Fractions were collected and analysed with HPLC, and those fractions containing HMOs were combined.

### Isolation of bifidobacteria from infant faeces

Faecal homogenates were diluted 10^5^–10^8^-fold with sterile saline (0.85% NaCl) and spread on bifidobacteria-specific TOS propionate agar plates (Yakult Honsha Co., Tokyo, Japan) supplemented with 50 mg l^−1^ mupirocin. The plates were incubated at 37 °C for 3 days in an anaerobic chamber (Coy Laboratory Products, Grass Lake, MI, USA) with 88% N_2_, 5% CO_2_ and 7% H_2_. All resulting colonies appearing on the plates inoculated with the highest and second-highest dilutions of faecal homogenate (at least 10 colonies per plate) were then streaked on GAM agar plates (Nissui Seiyaku, Tokyo, Japan). These isolates were further classified by random amplified polymorphic DNA profiling, as described in ref. 53. Isolates with identical random amplified polymorphic DNA patterns using two primers (p1254, 5′- CCGCAGCCAA -3′; p1281, 5′- AACGCGCAAC -3′) were regarded as identical strain. Each strain was grown overnight in GAM broth (Nissui Seiyaku), centrifuged, suspended in GAM broth with 20% glycerol and stored at −80 °C. For further analysis, the strains were passaged twice in GAM broth and grown in an anaerobic chamber.

### *In vitro* growth of bifidobacterial strains in the presence of HMO as the sole carbon source

The bacterial strains listed in Fig. 4a were cultured in PY medium (200 mM PIPES, pH 6.7, 2 g l^−1^ Peptone, 2 g l^−1^ BBL Trypticase Peptone, 2 g l^−1^ Bacto Yeast Extract, 8 mg l^−1^ CaCl_2_, 19.2 mg l^−1^ MgSO_4_.7H_2_O, 80 mg l^−1^ NaCl, 4.9 mg l^−1^ hemin, 0.5 g l^−1^ L-cysteine hydrochloride and 100 ng l^−1^ vitamin K_1_), which was supplemented with lactose or HMOs. The PY medium (200 μl) was inoculated with bifidobacterial strains growing at an exponential phase (equivalent to OD_600_=5 × 10^−4^) and covered with 50-μl sterile mineral oil to prevent evaporation. Growth was monitored by measuring the OD_600_ using a PowerWave 340 plate reader (BioTek, Winooski, VT, USA) every 30 min in the anaerobic chamber. Two technical replicates were performed for each strain. The supernatants of the bacterial cultures were collected at the end of the exponential growth phase (40 h) and stored at −80 °C for subsequent glycoprofiling.

### Glycoprofiling of HMOs

Oligosaccharides in bacterial supernatants or faecal suspensions were labelled with an ultraviolet light-absorbing compound, *p*-aminobenzoic acid ethyl ester (ABEE), and quantified using HPLC, as described in ref. 26. Briefly, 200 μl of bacterial culture or faecal homogenate was used as the specimen, and arabinose was added as an internal standard (1 mM final concentration). The cultured media were mixed with 800 μl of chloroform:methanol (2:1) and stirred vigorously for 1 min. The upper layer was collected after centrifugation at 9,000*g* for 10 min and concentrated by evaporation at 70 °C under a nitrogen gas flow. The crude oligosaccharides were dissolved in 200 μl of distilled water. A 10- μl portion of this solution was added to 40 μl of freshly prepared reagent mixture (35 mg ABEE, 3.5 mg NaBH_3_CN, 41 μl acetic acid and 350 μl methanol). The mixtures were heated at 80 °C for 45 min in screw-capped vials, after which 500 μl of water was added. The mixture was extracted three times with 500 μl of diethyl ether to remove excess ABEE. The aqueous layer was evaporated, and the residue was dissolved in 300 μl of distilled water. Ten-microlitre samples of labelled HMOs were then separated on a Shimadzu Prominence HPLC System (Kyoto, Japan) with an L-column 2 ODS (Chemicals Evaluation and Research Institute, Japan). Fractions were eluted with an 87:13 (vol:vol) mixture of 100 mM ammonium acetate buffer (pH 4.5) and acetonitrile, using flow rates of 1.0 ml min^−1^ (0–33 min) and 2.0 ml min^−1^ (33–55 min) at 40 °C. Labelled oligosaccharides were detected with an SPD-20 A ultraviolet detector (Shimadzu) at 304 nm. Standard curves for the major HMO components were generated using solutions containing both the internal standard (1 mM arabinose) and oligosaccharides at varying concentrations. The peak areas of the saccharides detected were standardized and quantified by comparison with the internal standard. 3-fucosyllactose, lacto-*N*-fucopentaose (LNFP) I, lacto-*N*-difucohexaose (LNDFH) I and LNDFH II were purchased from Dextra Laboratories (Reading, UK). 2'-FL, DFL, LNT, lacto-*N*-neotetraose, LNFP II and LNFP III were purchased from IsoSep AB (Tullinge, Sweden). *N*-acetyl glucosamine was purchased from Nacalai Tesque (Kyoto, Japan). These structures are illustrated in Supplementary Fig. 12.

### Genome sequencing

The draft genomes of 29 bifidobacterial strains were sequenced with the MiSeq sequencing platform (Illumina, San Diego, USA). Multiplexed shotgun libraries were constructed using the Nextera XT DNA Sample Prep Kit v2 (Illumina). The resulting paired-end sequence reads (250 bp × 2) were assembled into contigs using the assembly software ABySS 1.3.5 (ref. 54). The optimal k-mer size was predicted using VelvetK (http://bioinformatics.net.au/software.velvetk.shtml; maximum value=128). To identify adjacent gene of fucosidase (GH29 and 95) and *FL-SBP*, draft contigs were aligned to the published genome of *B. breve* UCC2003 (Accession Number CP000303)^55^ or *B. longum* ss. *infantis* ATCC 15697 (CP001095)^22^ using BLAST to predict the order and orientation. Oligonucleotide primers were designed to anneal to each end of the neighbouring contigs, and PCR amplification was performed to confirm the contig ordering. The resulting amplicons were subjected to Sanger sequencing. Draft genome sequences of 29 bifidobacterial strains were deposited in the DDBJ whole-genome shotgun database under BioProject Accession No. PRJDB4041.

### Annotation and comparative genome analysis

Protein-coding sequences (CDSs) on >500-bp contigs were predicted using the gene prediction software Glimmer 3.02 (ref. 56). Functional annotations of CDSs were performed by BLASTP searches (version 2.2.24)^57^ against the KEGG database^58^, with an *e*-value cutoff of 10^−6^. All-against-all BLASTP searches for all proteins from the 29 strains were performed with an *e*-value cutoff of 10^−5^ and a minimum identity of 55%, and the results were clustered using the OrthoMCL method^59^. rRNA genes were identified by BLASTN analysis using known bifidobacterial rRNA sequences as queries. The genes annotated as fucosidase (both GH29 and GH95) by BLASTP searching were aligned using Clustal X (ref. 60) and were used as inputs for calculating phylogenetic relationships. Phylogenetic trees were computed with the neighbour-joining method using Clustal X and were visualized using FigTree (http://tree.bio.ed.ac.uk/software/figtree; Supplementary Fig. 17). Localization of the fucosidase proteins was predicted based on their amino-acid sequences using the PSORT software (http://psort.hgc.jp/form.html).

### Multilocus sequence analysis

Identification of isolated strains by multilocus sequence analysis was performed by targeting seven reference genes (16S rRNA, *clpC, fusA, groEL, gyrB1, purF* and *xfp*). The genome sequences of *B. breve* UCC2003 (Accession Number CP000303) were used as queries for sequence identity searches, as described in ref. 61. The homologue sequences of the target genes were extracted from the 29 bifidobacterial strains by BLAST, concatenated and aligned with Clustal X (ref. 60). Phylogenetic trees were computed and visualized as described above.

### Construction of FL-SBP insertion mutant in *B. breve* BR-A29

The insertional mutagenesis procedure used to inactivate the putative FL-SBP gene in *B. breve* BR-A29 is summarized in Supplementary Fig. 18. Briefly, a DNA fragment of the *E. coli* and *Bifidobacterium*-derived shuttle vector pBEΔ26 (generated from pBEΔ4 (ref. 62) by replacing the erythromycin-resistance gene *ermE* with the tetracycline-resistance gene *tetW*) was generated by PCR amplification using KOD-plus-DNA polymerase (Toyobo, Osaka, Japan). Amplification proceeded via the following primers: SacI0001F (5′- TCCGAGCTCCAGGTGGCACTTTTCGGGGAAATG -3′) and Kpn4067R (5′- GGGGTACCCCAAGGAAATGGCTATCAACGGTA -3′), where the underlined sequences represent *Sac*I and *Kpn*I restriction sites, respectively. To construct the p15aAT-KS plasmid, the PCR product was digested with *Sac*I and *Kpn*I, and ligated with multiple cloning site of pBluescript II KS(+) (Agilent Technologies, CA, USA). To construct the p15aAT-FL-SBP-int plasmid, an internal 1,000-bp fragment of FL-SBP (FL-SBP-int) was amplified from *B. breve* BR-A29 genomic DNA with the primers FL-SBP-Bam0194F (5′- CGGGATCCTGGAGCCTACCGTCAAGGCATTCGA -3′) and FL-SBP-Bam1193R (5′- CGGGATCCTCGAAATAGACCGAGTTAACGTCCG -3′). This amplicon was digested with *Bam*HI and ligated into p15aAT-KS. Thereafter, p15aAT-FL-SBP-int (or the control plasmid, pBEΔ26) was introduced into *B. breve* BR-A29 by electroporation (18 kV cm^−1^, 200 Ω, 25 μF), and tetracycline-resistant transductants were selected. Site-specific recombination was confirmed by colony PCR with the FL-SBP-0078 F (5′- CGACACTAACGGAAGCCAGGCTA -3′) and ATKS-0097R (5′- CAGGGTTATTGTCTCATGAGCGGATAC -3′) primers.

### Data availability

Infant gut 16S rRNA gene microbiome data have been deposited in the DDBJ Sequence Read Archive (DRA) under BioProject accession code PRJDB4038. The draft genome sequences of the 29 bifidobacterial strains have been deposited in the DDBJ whole-genome shotgun database under BioProject accession code PRJDB4597. The FL-SBP gene sequence of *B. breve* BR-A29 has been deposited in the DDBJ database under accession code LC068768. The authors declare that all other data supporting the findings of this study are available within the article and its Supplementary Information Files, or from the corresponding author upon request.

## Additional information

**How to cite this article:** Matsuki, T. *et al*. A key genetic factor for fucosyllactose utilization affects infant gut microbiota development *Nat. Commun.* 7:11939 doi: 10.1038/ncomms11939 (2016).

## Supplementary Material

Supplementary Information Supplementary Figures 1-20 and Supplementary Tables 1-7

Supplementary Data 1 Information regarding subject backgrounds, bacterial abundances (%), α-diversities, PC1 and PC2 values shown in Figure 1c, microbiota cluster, and 454-sequence BioSample ID deposited in DDBJ DRA database.

Supplementary Data 2 Bacterial abundances (%), α-diversities, bacterial counts per g of faeces, PC1 and PC2 values.

Supplementary Data 3 pH values and concentrations of organic acids and oligosaccharides in infant faeces.

## Figures and Tables

**Table 1:** Summary of the draft genomes of the 29 bifidobacterial strains.

**Strains**	**Genome size (Mbp)**	**No. of CDSs**	**Growth with** **HMO (OD**_**600**_**>0.7)**	**α-*****L-*****fucosidase**		**HG_2571**
				**GH29**	**GH95**	**SBP of ABC transporter**
*B. breve* BR-06, BR-10, BR-14, BR-15, BR-20, BR-21, BR-A29, BR-I29 (eight strains)	2.2–2.7	1,928–2,368	+	−	+	+
*B. breve* BR-07, BR-19, BR-C29, BR-H29, BR-L29 (five strains)	2.2–2.5	1,955–2,224	−	−	+	−
*B. longum* ss. *infantis* IN-07, IN-F29 (two strains)	2.6–2.7	2,356–2,441	+	+	+	+
*B. longum* ss. *longum* LO-06, LO-10, LO-21, LO-C29, LO-K29a, LO-K29b (six strains)	2.4–2.7	1,987–2,209	−	−	−	−
*B. pseudocatenulatum* CA-C29, CA-K29a, CA-K29b (three strain)	2.2–2.5	1,825–2,168	+	−	+	+
*B. pseudocatenulatum* CA-05, CA-B29, CA-D29 (three strains)	2.2–2.3	1,896–1,904	−	−	−	−
*B. bifidum* BI-14	2.2	1,779	+	+	+	−
*B. dentium* DE-29	2.6	2,127	−	+	−	−

HMO, human milk oligosaccharide; SBP, substrate-binding protein.
