# Supplementary material for: A key genetic factor for fucosyllactose utilization affects infant gut microbiota development
Source: Nat Commun. 2016 Jun 24;7:11939. doi: 10.1038/ncomms11939 (PMC4931012; doi:10.1038/ncomms11939)
Supplement: Supplementary Information — Supplementary Figures 1-20 and Supplementary Tables 1-7 [file ncomms11939-s1.pdf]

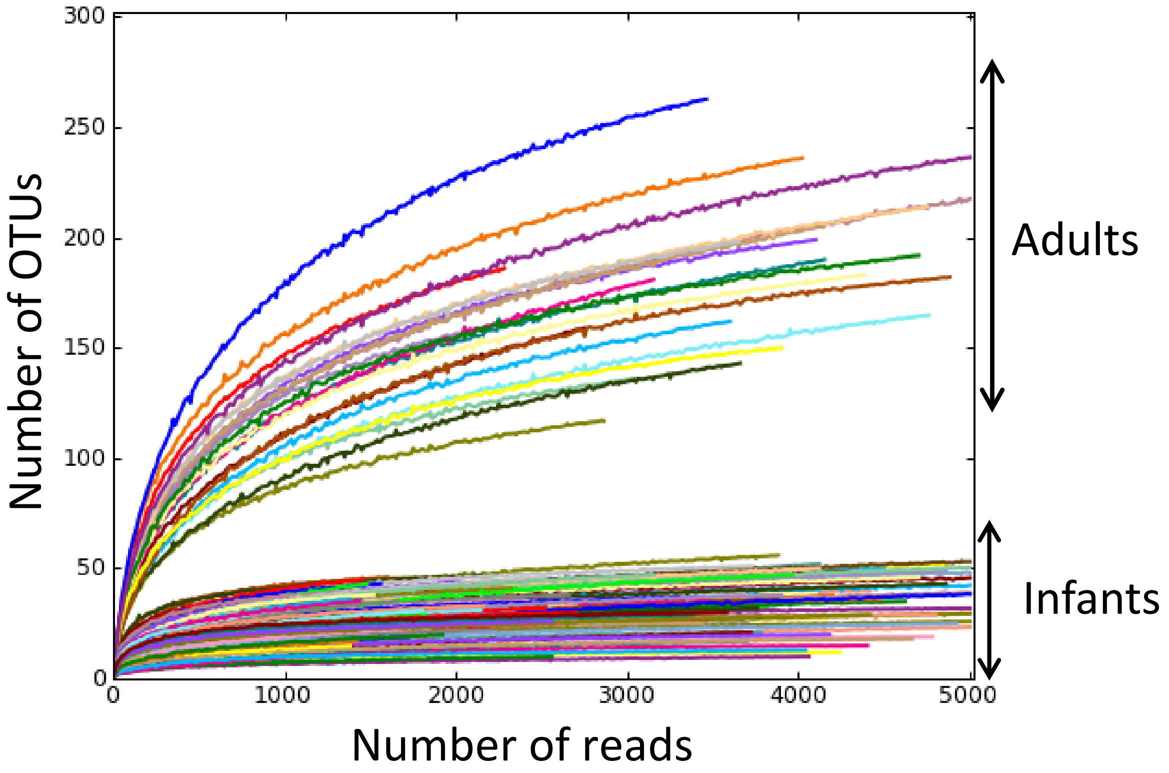

Supplementary Fig. 1. OTU-richness rarefaction curves of faecal microbiota evaluated in 217 stool samples from 27 infants and 22 stool samples from 22 adults.

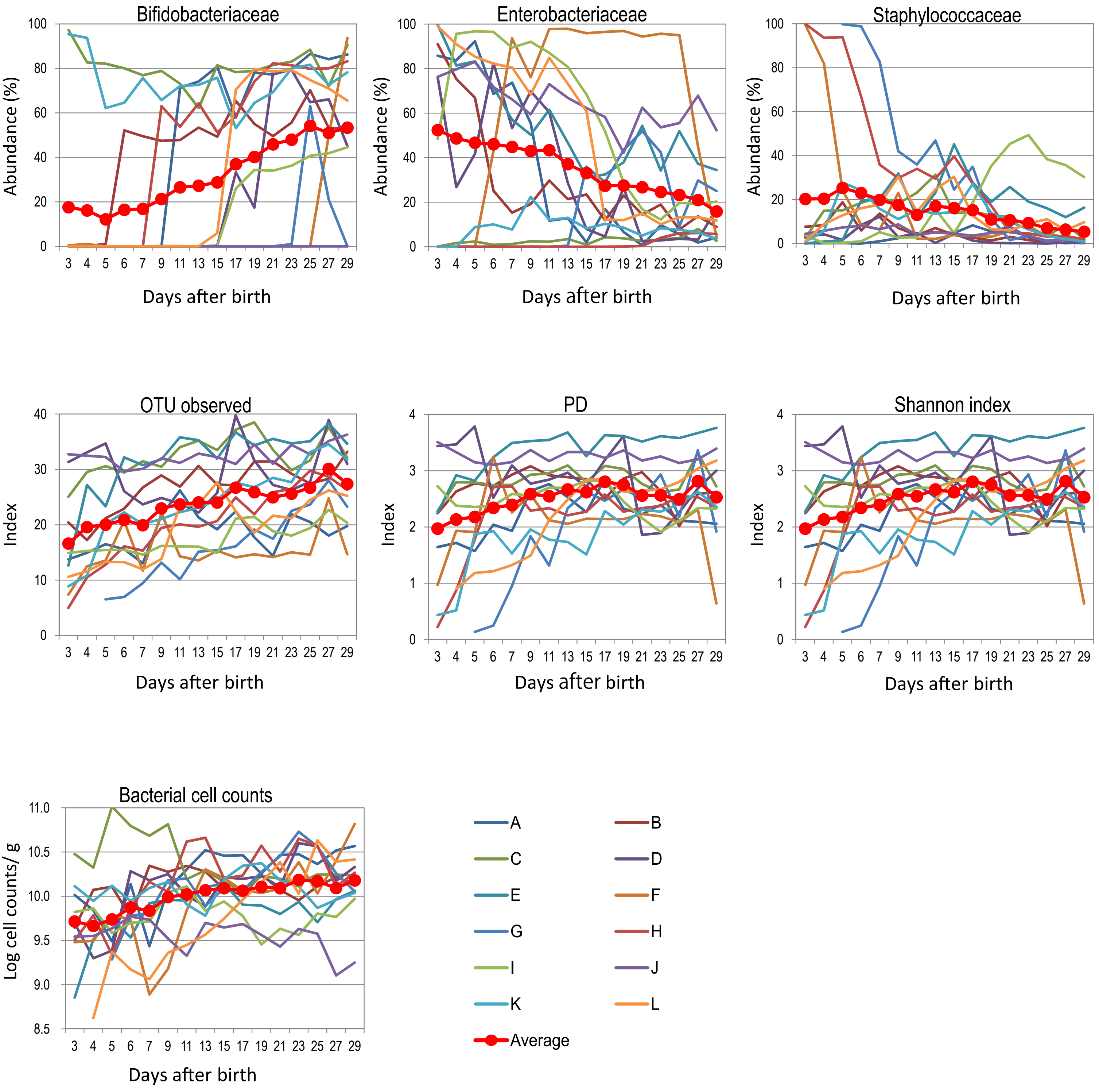

Supplementary Fig. 2. Changes in bacterial abundances, α-diversities, and total bacterial counts over the indicated days after birth. The values for each infant and the overall averages are shown.

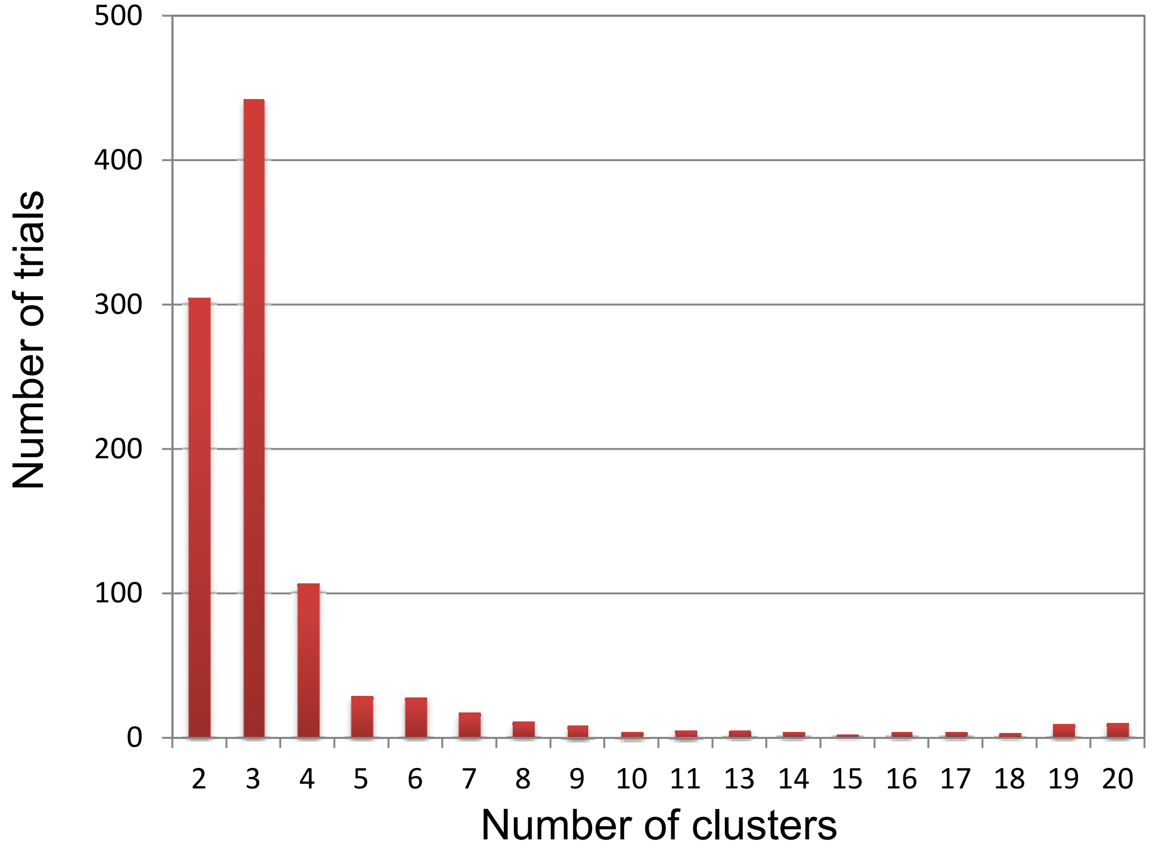

Supplementary Fig. 3. Estimation of the number of clusters in infant gut microbiota by PAM clustering. The number of clusters exhibiting the highest Calinski-Harabasz (CH) index was examined in the dataset of 100 randomly selected samples from a total of 202 samples (the trial was repeated 1,000 times), which indicate the presence of 3 distinct clusters in infant microbiota during 1st month of life.

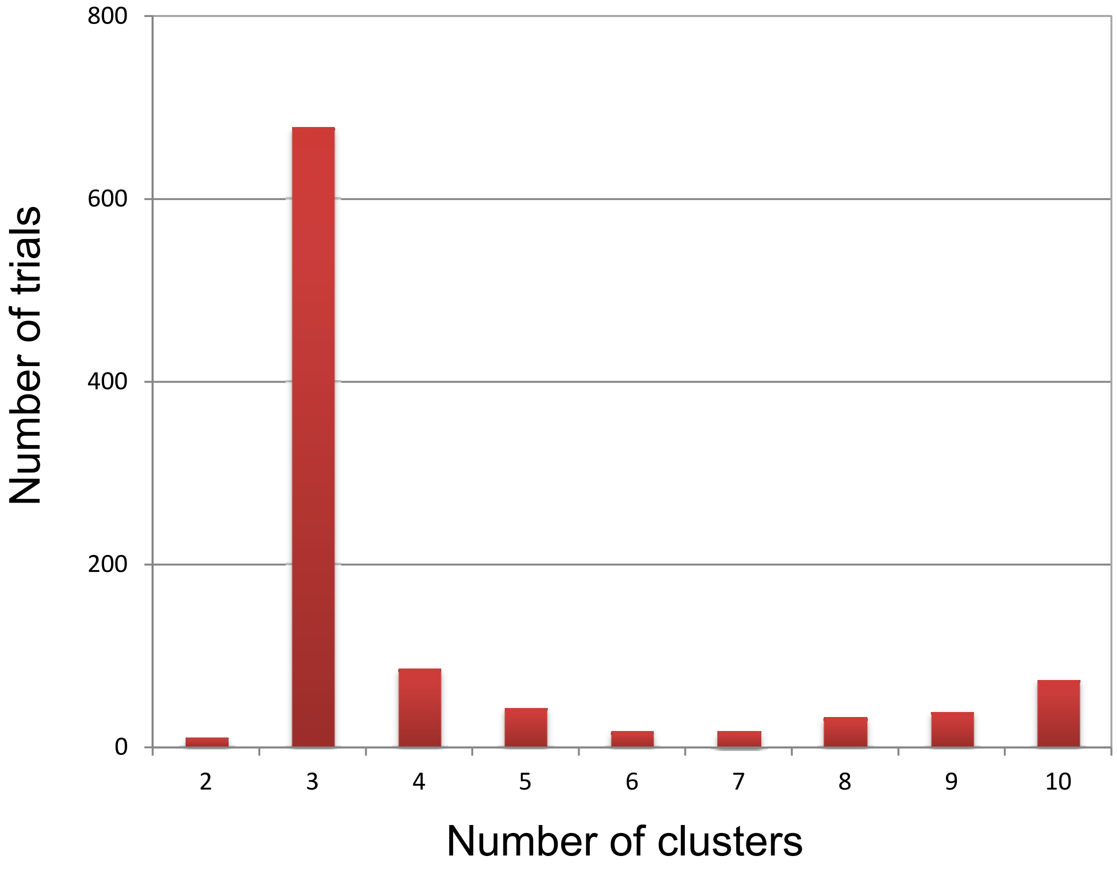

Supplementary Fig. 4. Estimation of the number of clusters in infant and adult gut microbiota by PAM clustering. The number of clusters exhibiting the highest Calinski-Harabasz (CH) index was examined in the dataset of 25 randomly selected samples from a total of 49 samples (the trial was repeated 1,000 times), which indicate the presence of 3 distinct clusters in microbiota of 1-month-old-infants and adults.

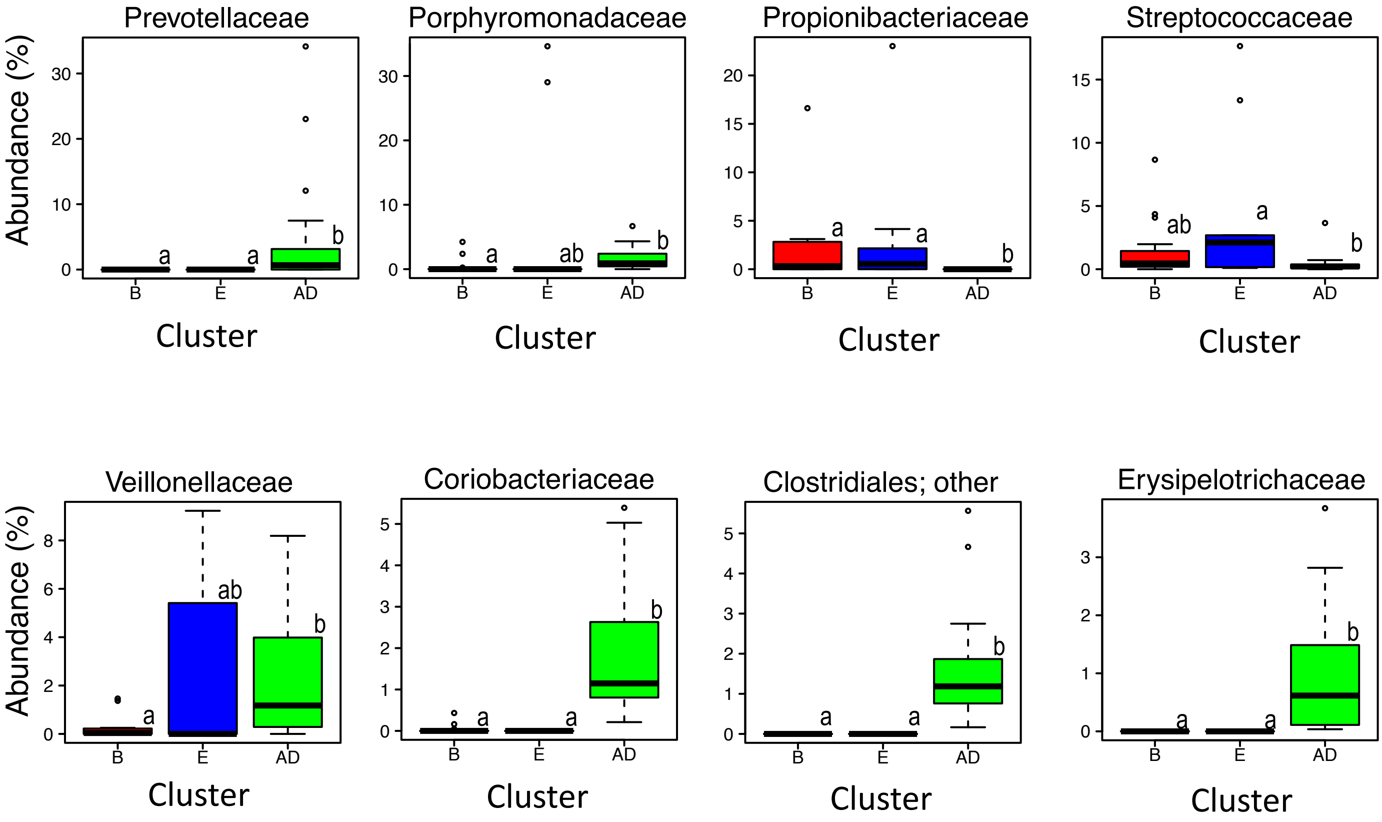

Supplementary Fig. 5. Differences in bacterial abundances among the clusters. Different letters (a–c) above the boxes indicate significant differences between groups ($p < 0.05$, Mann–Whitney U test with Bonferroni's correction). Cluster B (Bifidobacteriaceae-predominant, red), cluster E (Enterobacteriaceae-predominant, blue) and cluster AD (adult-type microbiota, green) were compared.

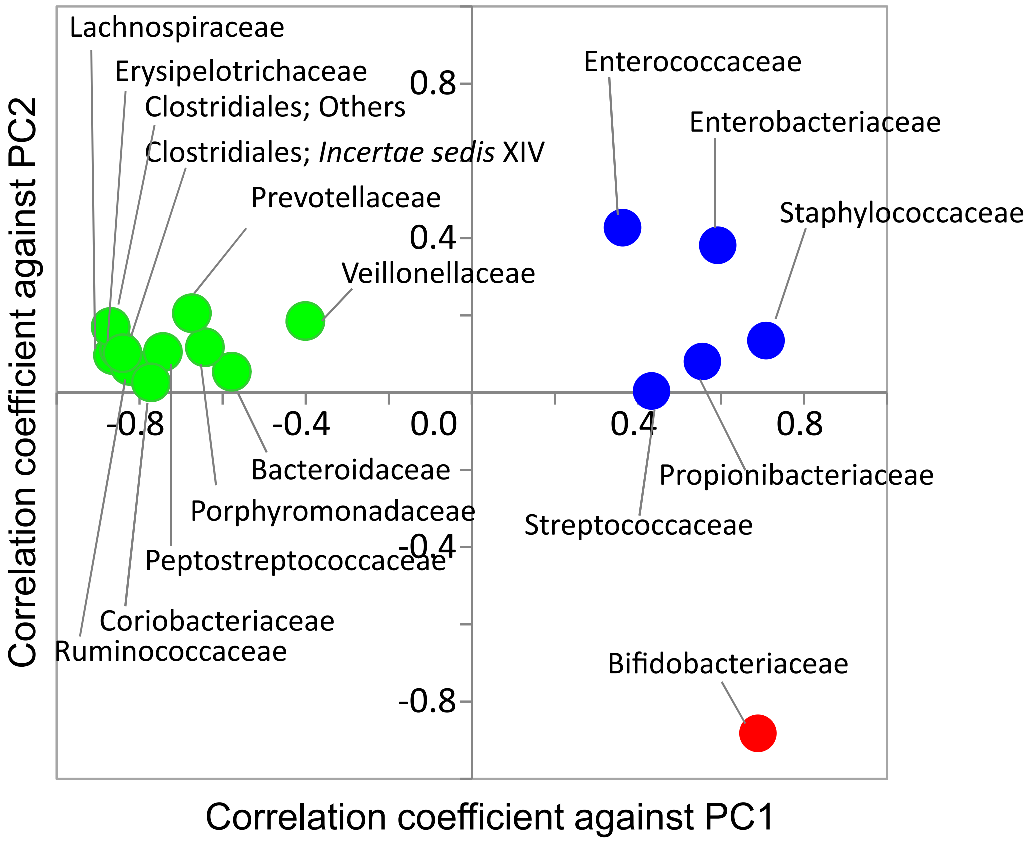

Supplementary Fig. 6. Plot of Spearman's correlation coefficients between the abundances of bacterial taxa and the PC axes. The contributors of cluster B (red), E (blue), and AD (green) with average population ≥1% were shown.

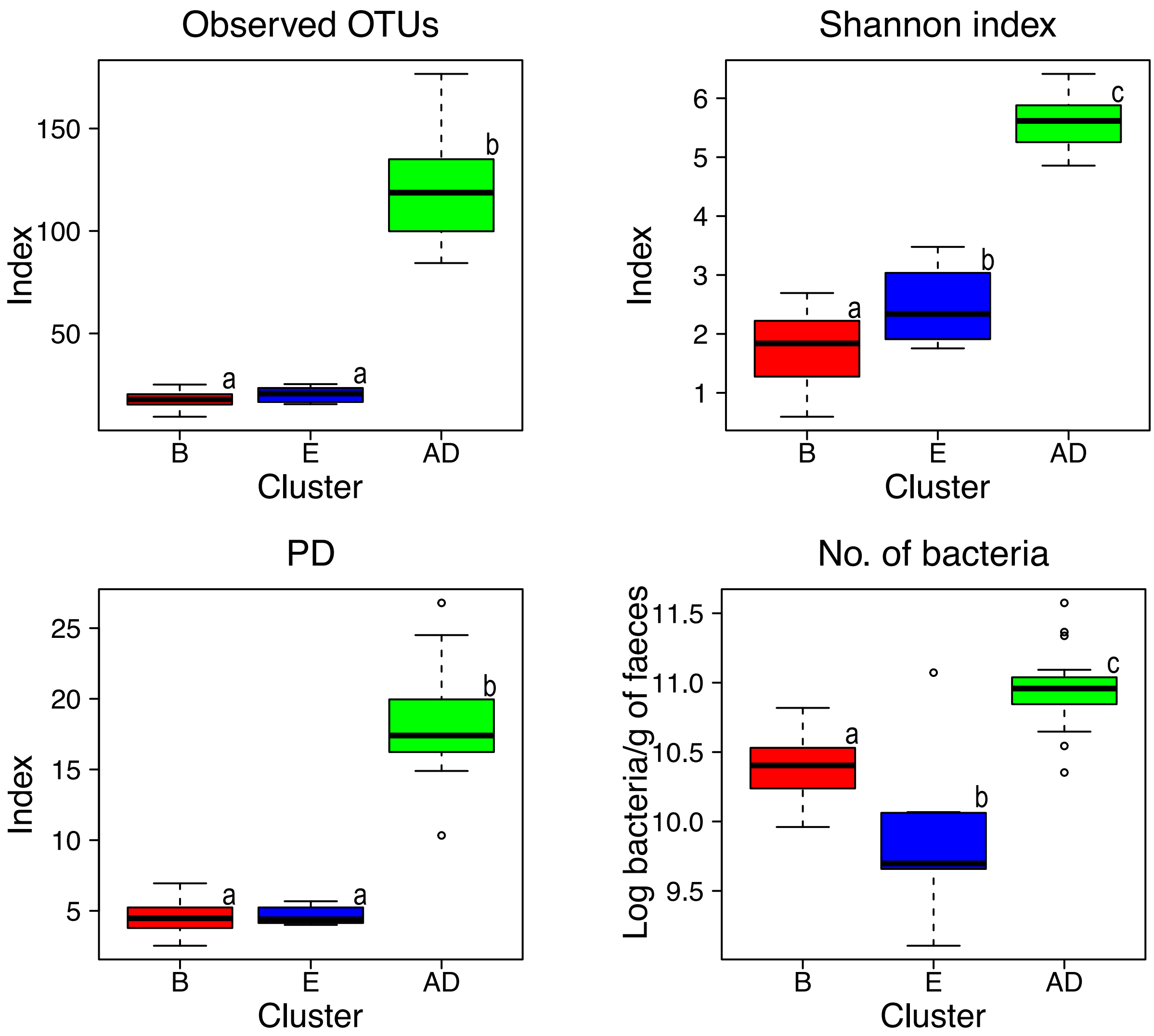

Supplementary Fig. 7. Differences in α-diversities and the numbers of bacteria among the clusters. Different letters (a–c) above the boxes indicate significant differences between groups ($p < 0.05$, Mann–Whitney U test with Bonferroni's correction). Cluster B, (Bifidobacteriaceae-predominant, red), cluster E (Enterobacteriaceae-predominant, blue) and cluster AD (adult-type microbiota, green) were compared.

Bacterial family	Average ± SD	Detected (%)	Bacterial abundance (%)										No. of bacteria (log)	α-diversity		
			Bifidobacteriaceae	Enterobacteriaceae	Enterococcaceae	Bacteroidaceae	Staphylococcaceae	Porphyromonadaceae	Streptococcaceae	Propionibacteriaceae	Veillonellaceae	Clostridiaceae		Observed OTUs	PD	Shannon index
Bifidobacteriaceae	53.9 ± 39.4	70.4		-0.91	-0.63	-0.02	-0.42	-0.10	-0.21	-0.23	-0.05	-0.53	0.67	-0.47	-0.24	-0.65
Enterobacteriaceae	21.3 ± 23.8	96.3			0.66	-0.12	0.38	-0.07	0.29	0.16	0.13	0.46	-0.67	0.19	0.08	0.45
Enterococcaceae	5.1 ± 14.8	55.6				-0.21	0.15	0.05	0.06	-0.04	-0.01	0.13	-0.32	0.06	-0.09	0.19
Bacteroidaceae	4.4 ± 9.0	33.3					-0.19	0.64	-0.40	-0.36	-0.23	0.10	-0.15	0.56	0.27	0.34
Staphylococcaceae	4.2 ± 7.6	92.6						-0.04	0.56	0.07	0.20	0.38	-0.61	0.23	0.13	0.49
Porphyromonadaceae	2.6 ± 8.5	18.5							-0.22	-0.17	-0.25	-0.08	-0.19	0.54	0.32	0.35
Streptococcaceae	2.4 ± 4.3	96.3								0.03	0.54	0.15	-0.24	0.03	0.27	0.07
Propionibacteriaceae	2.4 ± 5.3	59.3									0.05	0.23	0.00	0.08	0.24	0.19
Veillonellaceae	1.1 ± 2.4	55.6										-0.01	-0.24	-0.01	0.37	0.01
Clostridiaceae	1.1 ± 2.6	33.3											-0.27	0.40	0.44	0.54
No. of bacteria (log)	10.2 ± 0.4	-												-0.43	-0.14	-0.53
Observed OTUs	18.5 ± 4.2	-													0.63	0.75
PD	4.6 ± 0.9	-														0.43
Shannon index	2.0 ± 0.7	-														

Correlation coefficients				
-1.0	-0.5	0.0	0.5	1.0

Supplementary Fig. 8. Correlation matrix for the 27 1-month-old infants in this study. Spearman’s rank correlation coefficients between bacterial family abundances (average; ≥1%), numbers of bacteria, and α-diversities are shown numerically and in colour-scale. Underlining indicates p values < 0.05. Of the 45 values, 11 were statistically significant in infants.

Bacterial family	Average ± SD	Detected (%)	Bacterial abundance (%)														No. of bacteria (log)	α-diversity		
			Lachnospiraceae	Clostridiales (XIV)	Bifidobacteriaceae	Ruminococcaceae	Bacteroidaceae	Prevotellaceae	Peptostreptococcaceae	Veillonellaceae	Enterobacteriaceae	Coriobacteriaceae	Porphyromonadaceae	Clostridiales unclasified	Eubacteriaceae	Erysipelotrichaceae		Observed OTUs	PD	Shannon index
Lachnospiraceae	30.9 ± 12.1	100		0.17	-0.21	-0.14	-0.24	-0.20	-0.07	-0.48	-0.44	-0.17	-0.44	0.05	-0.55	0.02	-0.37	-0.26	-0.26	-0.09
Clostridiales Incertae Sedis XIV	15.4 ± 7.3	100			-0.20	-0.03	-0.59	0.01	-0.31	-0.12	-0.27	0.07	-0.35	0.34	0.18	0.19	-0.09	0.06	0.13	0.03
Bifidobacteriaceae	14.3 ± 10.9	91				-0.28	-0.17	-0.58	0.14	-0.16	0.54	0.01	-0.28	-0.59	-0.17	-0.19	-0.17	-0.44	-0.21	-0.42
Ruminococcaceae	11.4 ± 5.3	100					0.30	0.06	0.20	0.42	-0.33	-0.08	0.34	0.19	0.05	0.23	0.60	0.70	0.65	0.70
Bacteroidaceae	7.4 ± 5.5	100						-0.01	0.36	0.00	-0.07	-0.47	0.52	-0.13	-0.04	-0.04	0.35	0.32	0.13	0.28
Prevotellaceae	4.3 ± 8.6	64							0.01	0.39	0.05	0.43	0.42	0.44	0.28	0.01	0.15	0.45	0.41	0.45
Peptostreptococcaceae	2.7 ± 3.4	95								0.36	-0.03	0.20	-0.13	-0.15	-0.06	0.36	-0.11	0.03	0.17	0.15
Veillonellaceae	2.2 ± 2.5	91									-0.18	0.42	0.22	0.08	0.51	0.45	0.23	0.44	0.40	0.43
Enterobacteriaceae	1.9 ± 5.6	59										0.14	0.02	-0.25	-0.06	-0.38	-0.04	-0.25	-0.09	-0.28
Coriobacteriaceae	1.8 ± 1.5	100											-0.24	0.16	0.14	-0.02	-0.30	-0.08	0.08	-0.16
Porphyromonadaceae	1.6 ± 1.8	95												0.29	0.29	0.01	0.57	0.68	0.49	0.58
Clostridiales unclasified	1.6 ± 1.3	100													0.08	0.19	0.20	0.28	0.15	0.25
Eubacteriaceae	1.2 ± 3.3	73														0.31	0.21	0.34	0.31	0.17
Erysipelotrichaceae	1 ± 1.1	100															0.20	0.35	0.33	0.39
No. of bacteria (log)	11 ± 0.3	-																0.72	0.66	0.68
Observed OTUs	120 ± 23	-																	0.89	0.91
PD	18.2 ± 3.5	-																		0.81
Shannon index	5.6 ± 0.5	-																		

Correlation coefficients

-1.0	-0.5	0.0	0.5	1.0

Supplementary Fig. 9. Correlation matrix for 22 adult volunteers. Spearman's rank correlation coefficients between bacterial family abundances (average; ≥1%), total bacterial counts, and α-diversities are shown numerically and in color-scale values. Underlining indicates p values < 0.05. Of the 91 values, 16 were statistically significant in adults.

			Family abundance (%)											Organic acids (mM)							Faecal oligosaccharides (summary, mM)				Faecal oligosaccharides (detail, mM)							
	Average ± SD	% detected	Bifidobacteriaceae	Enterobacteriaceae	Enterococcaceae	Bacteroidaceae	Staphylococcaceae	Porphyromonadaceae	Streptococcaceae	Propionibacteriaceae	Veillonellaceae	Clostridiaceae	pH	Acetate	Lactate	Succinate	Propionate	Formate	Total organic acids	No. of bacteria (log)	Sum (FL)	Sum (LNT+LNnT)	Sum (LNFP+LNDFH)	Total HMO	2'-FL	3-FL	DFL	LNT	LNnT	LNFP	LNDFH I	LNDFH II
Bifidobacteriaceae	54 ± 39	70		-0.91	-0.63	-0.02	-0.42	-0.10	-0.21	-0.23	-0.05	-0.53	-0.53	0.47	0.39	-0.58	-0.20	0.74	0.36	0.67	-0.59	-0.48	-0.40	-0.56	-0.53	-0.68	-0.39	-0.53	0.00	-0.44	-0.28	0.04
Enterobacteriaceae	21 ± 24	96			0.66	-0.12	0.38	-0.07	0.29	0.16	0.13	0.46	0.59	-0.49	-0.32	0.50	0.24	-0.74	-0.39	-0.67	0.61	0.54	0.35	0.57	0.54	0.71	0.36	0.58	0.00	0.48	0.15	-0.04
Enterococcaceae	5.1 ± 14.8	56				-0.21	0.15	0.05	0.06	-0.04	-0.01	0.13	0.31	-0.25	-0.15	0.33	0.30	-0.30	-0.15	-0.34	0.35	0.33	0.20	0.34	0.38	0.40	0.29	0.42	-0.05	0.34	0.14	-0.15
Bacteroidaceae	4.4 ± 9	33					-0.19	0.64	-0.40	-0.36	-0.23	0.10	-0.43	0.29	-0.06	0.34	-0.07	-0.20	0.20	-0.14	-0.24	-0.29	-0.15	-0.31	-0.06	-0.26	-0.39	-0.34	-0.10	-0.20	-0.20	-0.10
Staphylococcaceae	4.2 ± 7.6	93						-0.04	0.56	0.07	0.20	0.38	0.38	-0.49	-0.13	0.02	-0.16	-0.38	-0.46	-0.58	0.20	0.11	0.39	0.32	0.30	0.32	0.08	0.13	0.03	0.32	0.35	0.23
Porphyromonadaceae	2.6 ± 8.5	19							-0.22	-0.17	-0.25	-0.08	-0.43	0.32	0.12	0.21	0.15	0.02	0.32	-0.23	-0.08	0.00	0.16	-0.04	-0.11	-0.10	-0.07	0.00	0.11	0.03	0.07	0.33
Streptococcaceae	2.4 ± 4.3	96								0.03	0.54	0.15	0.45	-0.22	0.09	-0.13	-0.14	-0.11	-0.18	-0.21	0.13	0.22	0.29	0.26	0.00	0.28	0.01	0.34	-0.14	0.28	0.17	0.13
Propionibacteriaceae	2.4 ± 5.3	59									0.05	0.23	0.46	-0.49	-0.60	-0.03	0.26	-0.28	-0.47	-0.03	0.46	0.31	0.38	0.51	0.19	0.33	0.53	0.18	0.45	0.35	0.44	0.31
Veillonellaceae	1.1 ± 2.4	56										-0.01	0.47	-0.23	-0.07	-0.01	0.28	-0.06	-0.23	-0.21	0.13	0.31	0.22	0.19	-0.11	0.31	0.02	0.34	-0.09	0.28	0.03	0.15
Clostridiaceae	1.1 ± 2.6	33											0.44	-0.35	-0.42	0.25	-0.09	-0.44	-0.33	-0.25	0.48	0.10	0.25	0.40	0.52	0.40	0.32	0.04	0.00	0.22	0.29	-0.05
pH	5.9 ± 0.6	-												-0.76	-0.43	0.09	0.15	-0.54	-0.73	-0.30	0.62	0.54	0.39	0.67	0.37	0.71	0.30	0.43	0.22	0.53	0.19	0.12
Acetate	30.5 ± 35.3	100													0.60	-0.04	-0.11	0.63	0.95	0.44	-0.72	-0.34	-0.60	-0.77	-0.55	-0.68	-0.27	-0.29	-0.32	-0.70	-0.46	-0.26
Lactate	12.9 ± 31	63														-0.11	-0.36	0.49	0.66	0.28	-0.53	-0.05	-0.40	-0.50	-0.41	-0.32	-0.31	-0.02	-0.23	-0.41	-0.39	-0.12
Succinate	8.9 ± 12	93															-0.04	-0.50	0.09	-0.28	0.18	0.19	0.04	0.09	0.23	0.32	0.18	0.12	-0.07	0.09	-0.07	-0.05
Propionate	1.1 ± 1.9	33																-0.05	-0.12	-0.20	0.22	0.25	0.36	0.26	0.02	0.29	0.32	0.36	0.06	0.33	0.32	0.19
Formate	1.3 ± 2.5	30																	0.57	0.58	-0.55	-0.30	-0.28	-0.52	-0.52	-0.66	-0.08	-0.22	-0.06	-0.42	-0.11	0.11
Total organic acids	54.7 ± 65.1	100																		0.40	-0.60	-0.21	-0.51	-0.65	-0.44	-0.54	-0.12	-0.14	-0.30	-0.63	-0.36	-0.25
No. of bacteria (log)	10.2 ± 0.4	-																			-0.41	-0.49	-0.46	-0.49	-0.39	-0.45	-0.17	-0.51	-0.21	-0.46	-0.26	-0.21
Sum (FL)	28.8 ± 28.2	100																				0.60	0.62	0.93	0.74	0.82	0.62	0.56	0.36	0.67	0.48	0.32
Sum (LNT+LNnT)	3.5 ± 5.2	100																					0.48	0.69	0.36	0.59	0.52	0.84	0.54	0.51	0.25	0.39
Sum (LNFP+FNDFH)	24.7 ± 17.5	100																						0.80	0.26	0.61	0.41	0.50	0.38	0.90	0.84	0.73
Total HMO	57 ± 41.7	100																							0.61	0.83	0.57	0.62	0.49	0.82	0.63	0.49
2'-FL	14.9 ± 20.8	78																								0.45	0.53	0.28	0.29	0.26	0.28	0.01
3-FL	10 ± 13.2	96																									0.40	0.59	0.13	0.72	0.43	0.21
DFL	3.9 ± 3.4	96																										0.46	0.39	0.21	0.54	0.36
LNT	3.2 ± 5.2	96																											0.16	0.53	0.34	0.24
LNnT	0.4 ± 0.5	67																												0.29	0.31	0.57
LNFP	13.2 ± 8.9	100																													0.61	0.57
LNDFH I	8.2 ± 8.7	93																														0.53
LNDFH II	3.3 ± 2.9	81																														

-1.0	-0.5	0.0	0.5	1.0

Supplementary Fig. 10. Expanded correlation matrix for 27 1-month-old infants. Spearman's rank correlations between the 10 most abundant bacterial families (average ≥1%), faecal pHs, organic acid concentrations, numbers of bacteria, and oligosaccharide concentration are shown numerically and in colour-scale. Underlining indicates p value < 0.05.

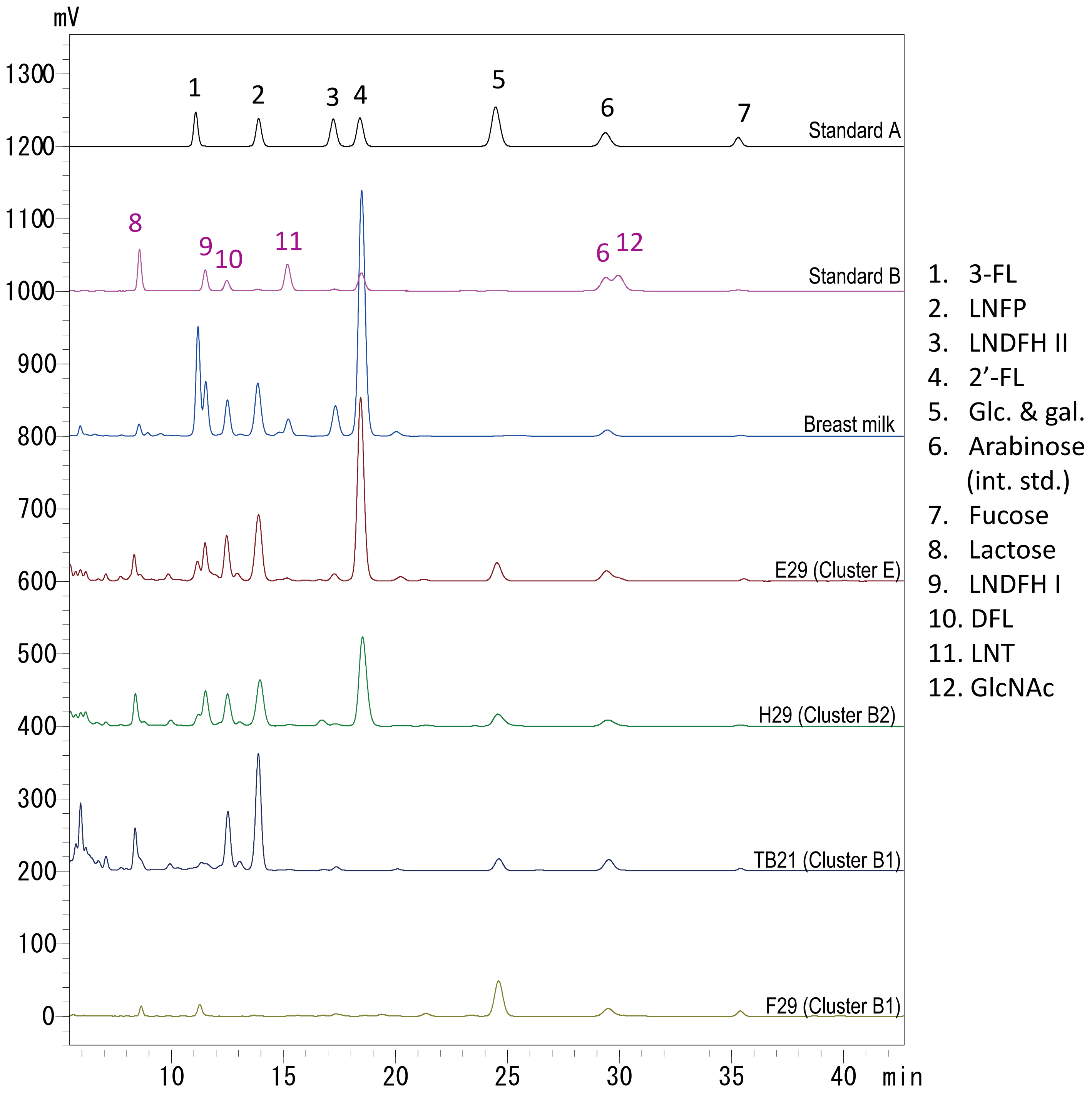

Supplementary Fig. 11. HPLC profiles of the remaining HMOs in infant stool samples. The sugars were labelled with ABEE and analysed by an HPLC instrument equipped with an L-column 2. Arabinose were used as an internal standard. The peaks were identified by comparing their retention times with those of the standard sugars (1–12).

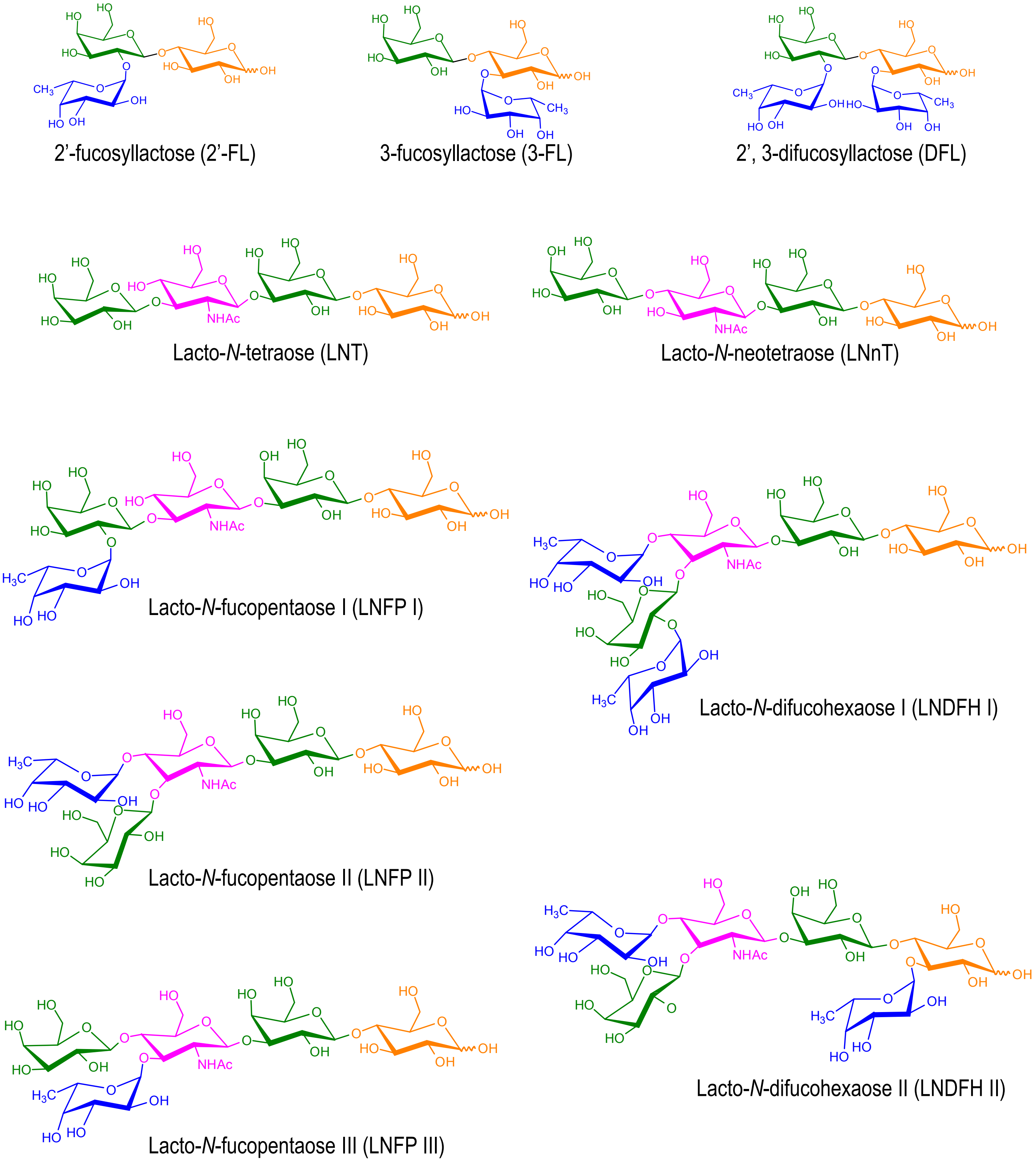

Supplementary Fig. 12. Chemical structures of the major HMO components analysed in this study.

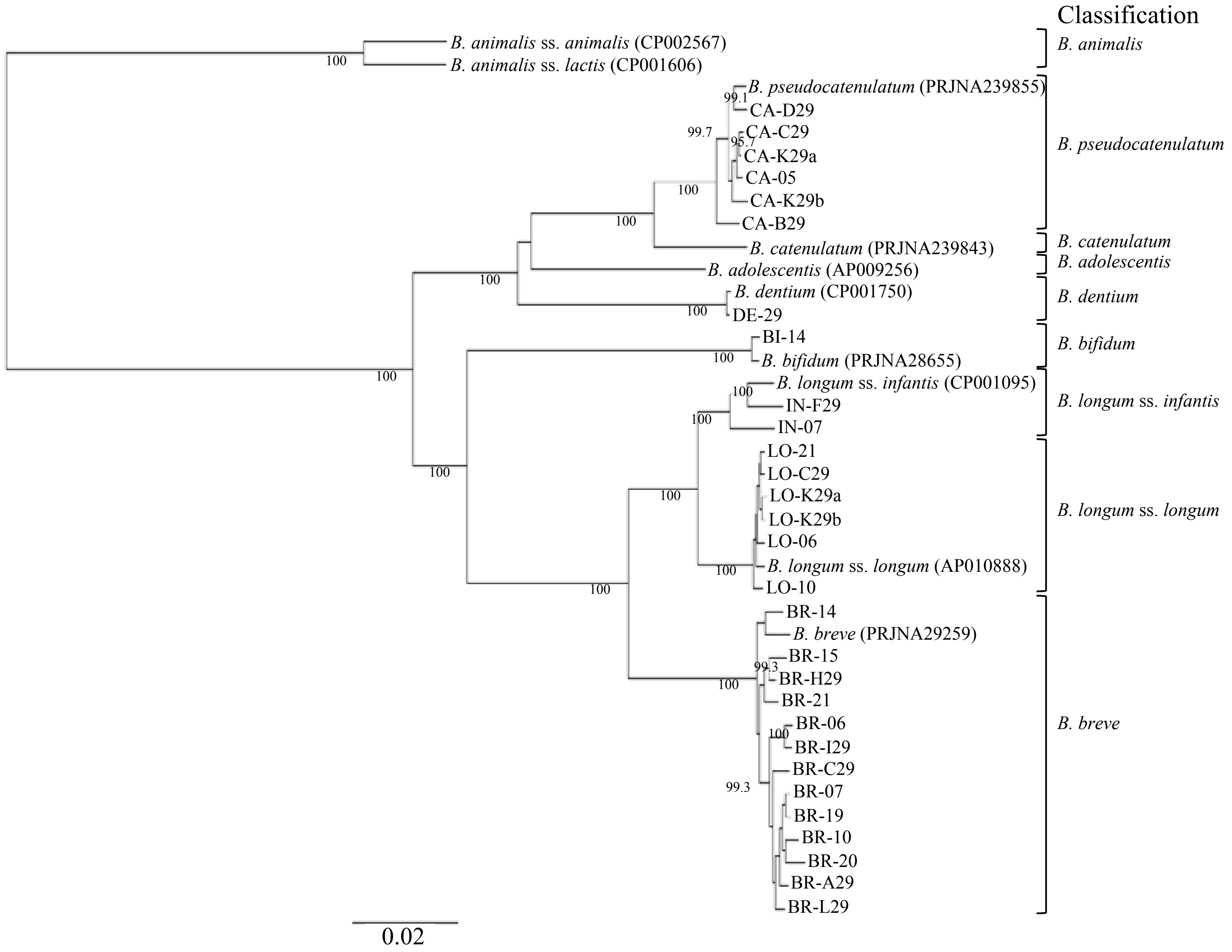

Supplementary Fig. 13. Identification of *Bifidobacterium* strains based on multilocus sequence analysis (MLSA). A phylogenic tree of 29 isolated strains was computed from the concatenation of 16S rRNA, *clpC, fusA, groEL, gyrB1, purF,* and *xfp* gene sequences by the neighbour-joining method. GenBank accession numbers of the reference strains are given in parentheses. Bootstrap values of the phylogenetic tree were computed by resampling 1000 times, with values >95% shown in the branches.

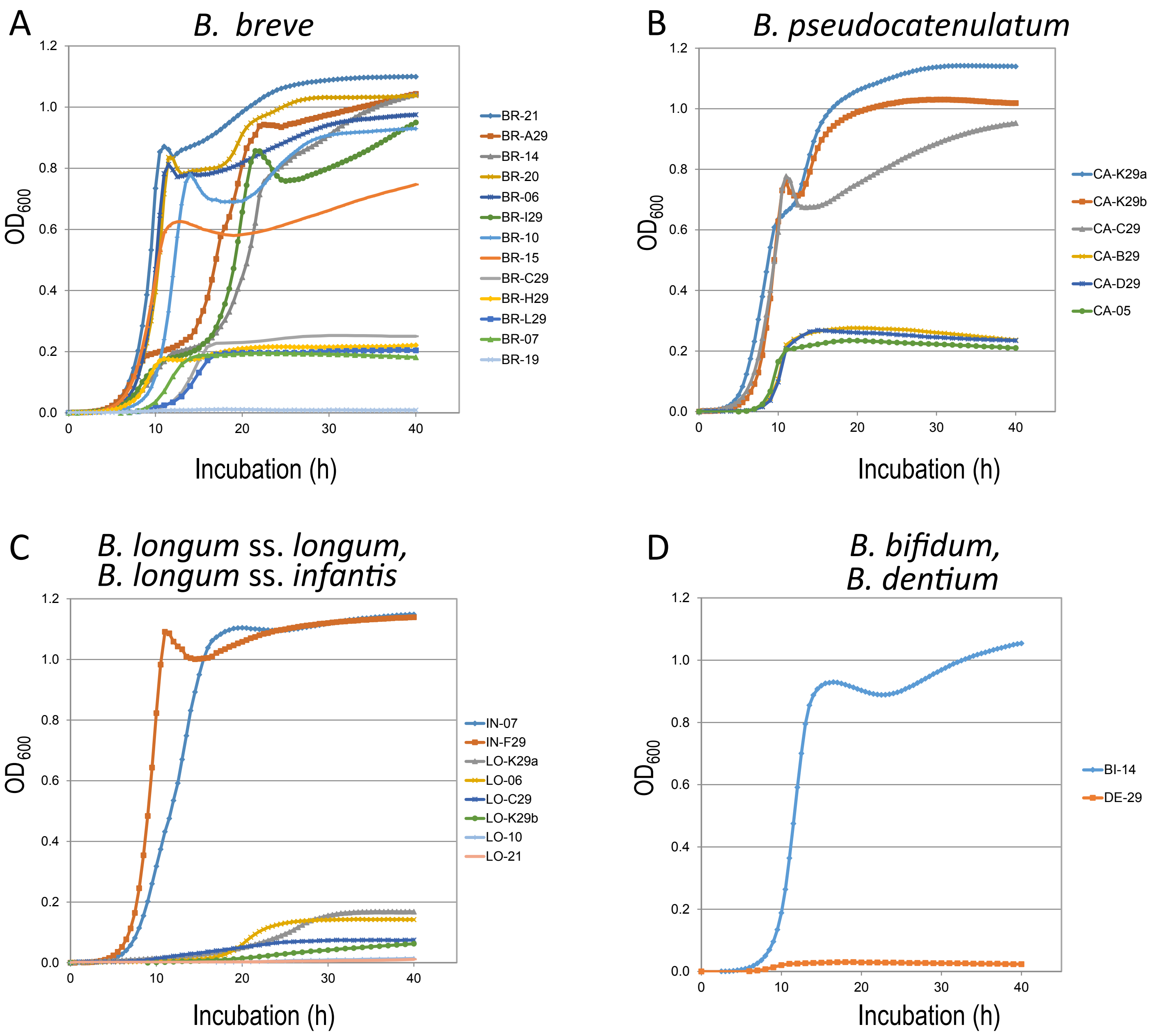

Supplementary Fig. 14. Growth curves of 29 bifidobacterial strains in medium containing HMOs.

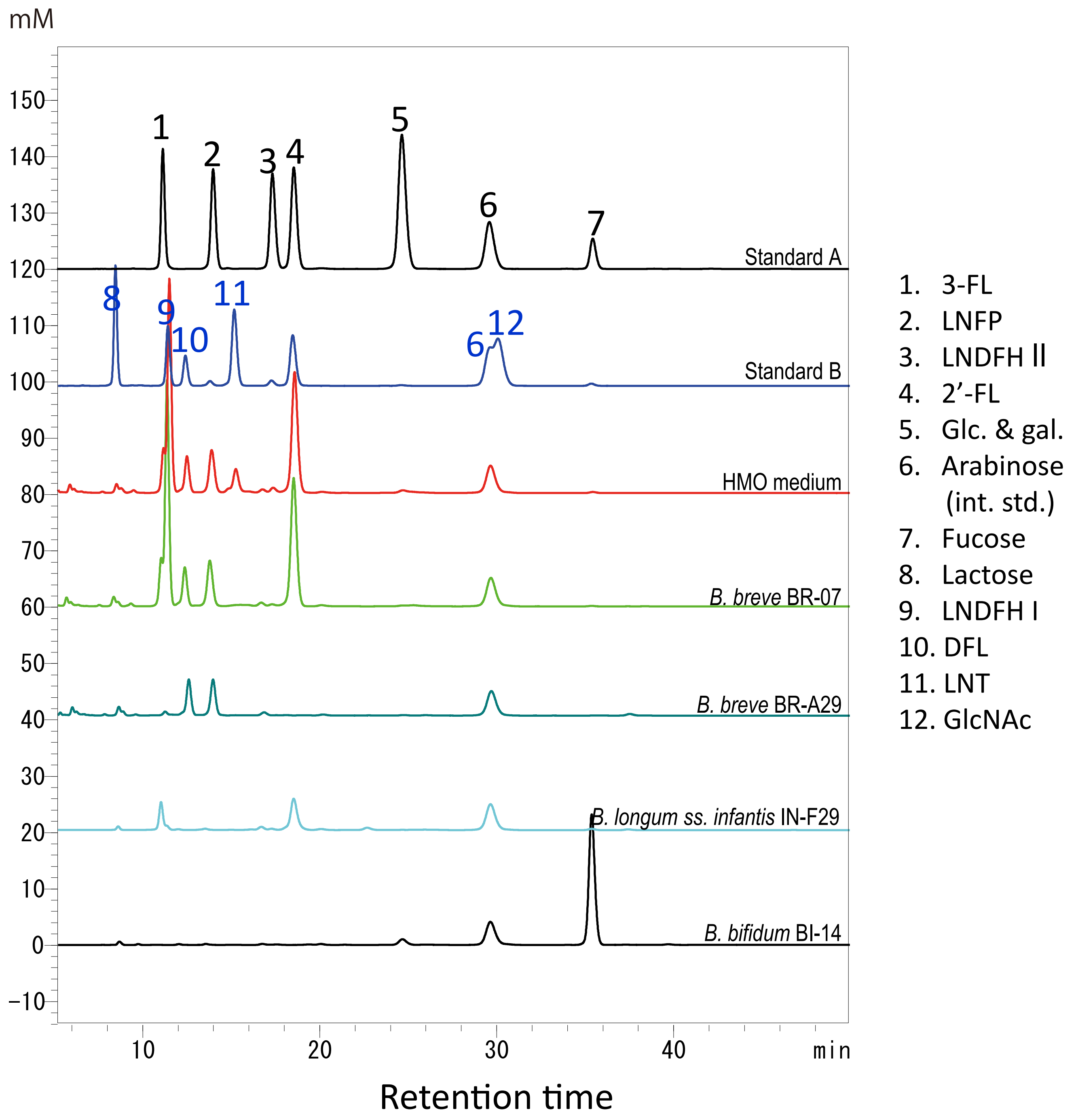

Supplementary Fig. 15. HPLC profiles of the remaining HMOs in bacterial culture supernatants after 40 h. The sugars were labelled with ABEE and analysed by HPLC. Arabinose was used as an internal standard. The peaks were identified by comparing their retention times with those of the standard sugars (1–12).

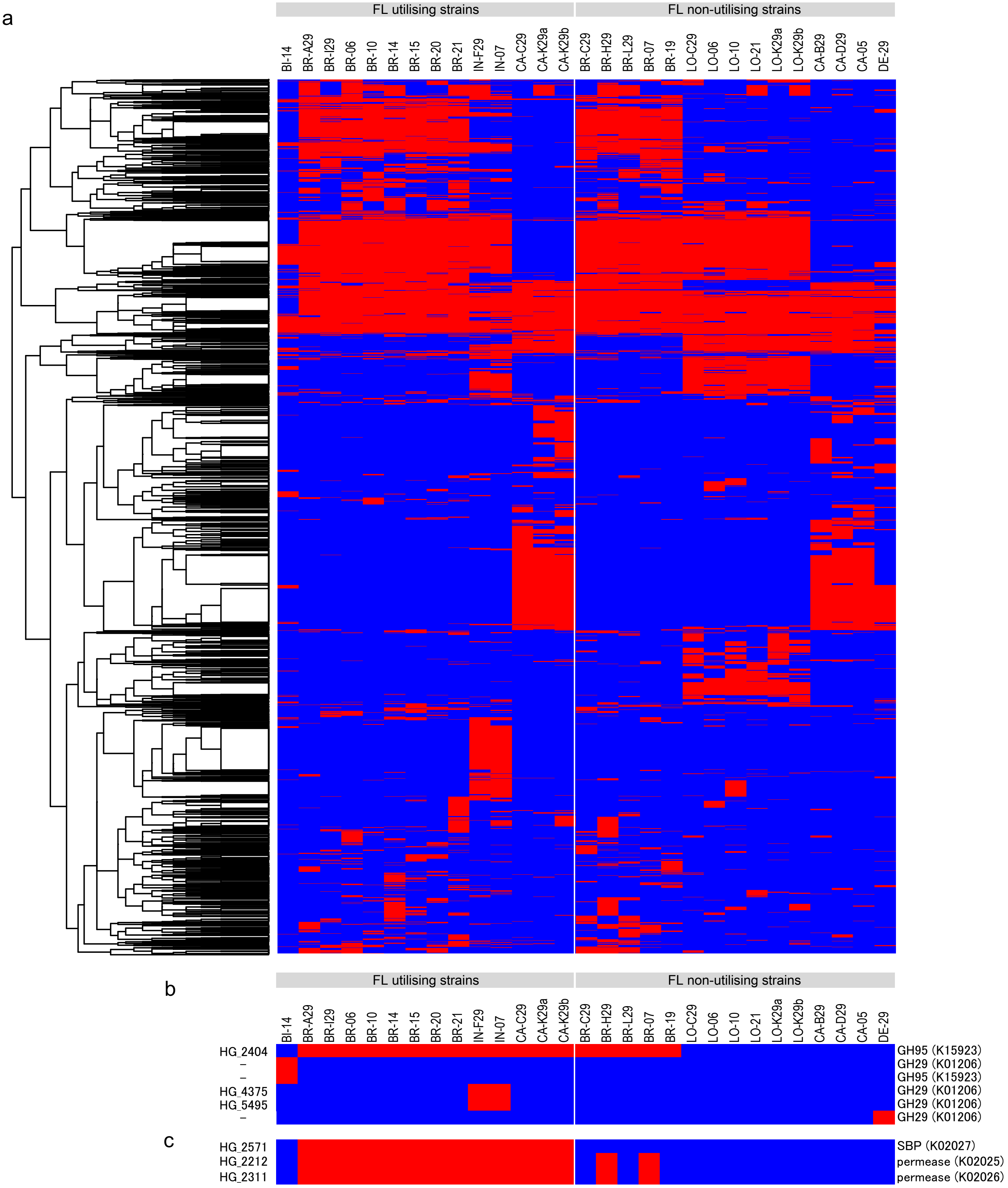

Supplementary Fig. 16. OrthoMCL clustering of 29 bifidobacterial strains based on bi-directional BLASTP searches. **a,** Among the 45,914 predicted genes of these strains, 4,690 homologous groups were identified, and their presence (red) or absence (blue) is shown. Similarity trees of OrthoMCL homologues (left) were computed based on the Euclidean distance of the homologue co-existent pattern.**b,** Distribution of homologous groups and a singleton assigned to fucosidases GH29 and GH95. **c,** Candidate genes responsible for FL utilisation. The presence of an homologous group (HG_2571) corresponded with the FL-utilisation phenotype in most strains.

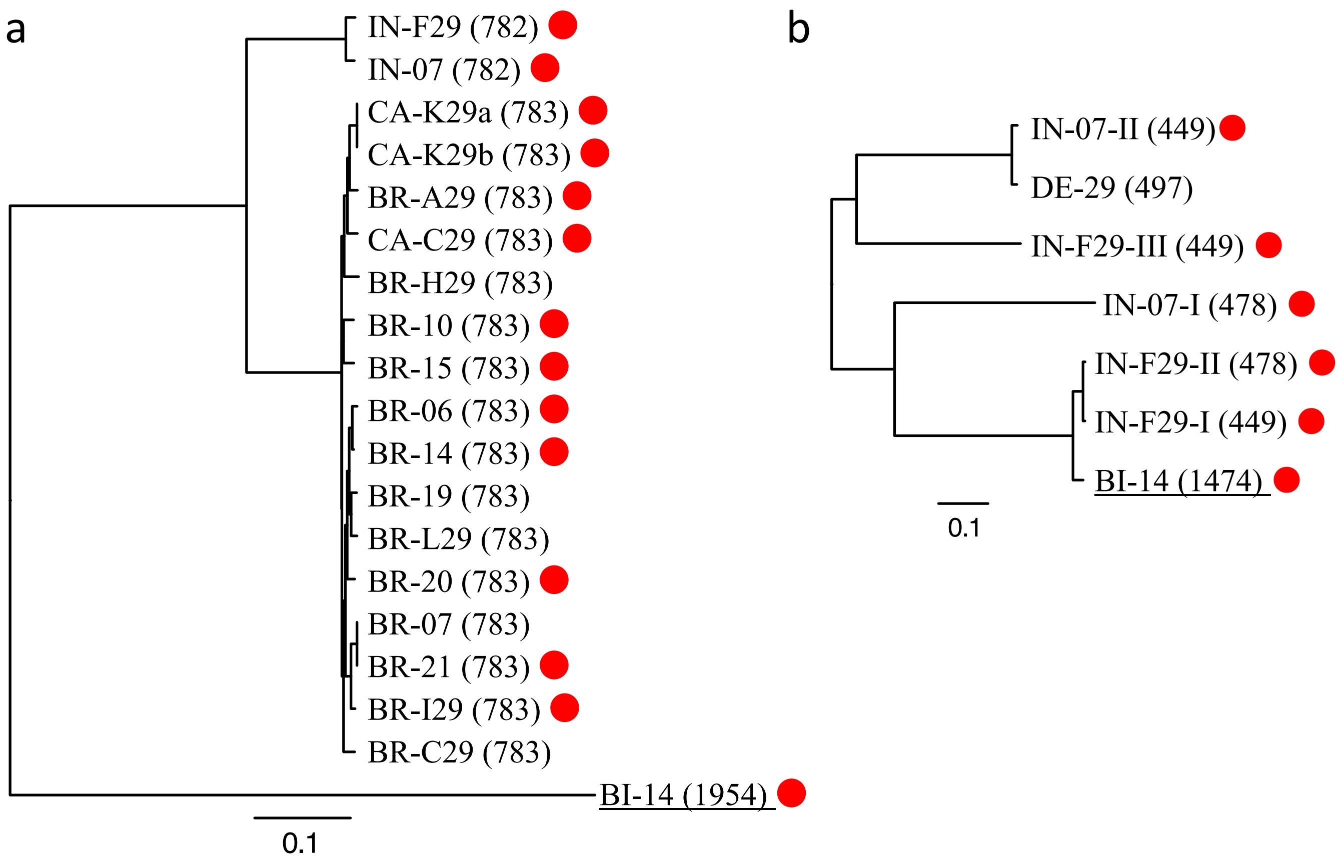

Supplementary Fig. 17. Phylogenetic tree of fucosidases of the isolated bifidobacteria. The strains labelled with a red circle are capable of utilising FL. The number in parenthesis represents the number of amino acids in the protein. Protein-localisation predictions suggested that most of these fucosidases are intracellular enzymes, except for the underlined fucosidase of the *B. bifidum* strain BI-14, which is predicted to be an extracellular membrane protein. **a,** Nineteen GH95 family genes found in 19 bifidobacterial strains. **b,** Seven GH29 family genes found in 3 strains. *B. longum* ss. *infantis* IN-F29 strain possess 3 GH29 genes (denoted as IN-F29-I, -II, and -III). IN-07 strain possess 2 fucosidase genes (IN-07-I and -II).

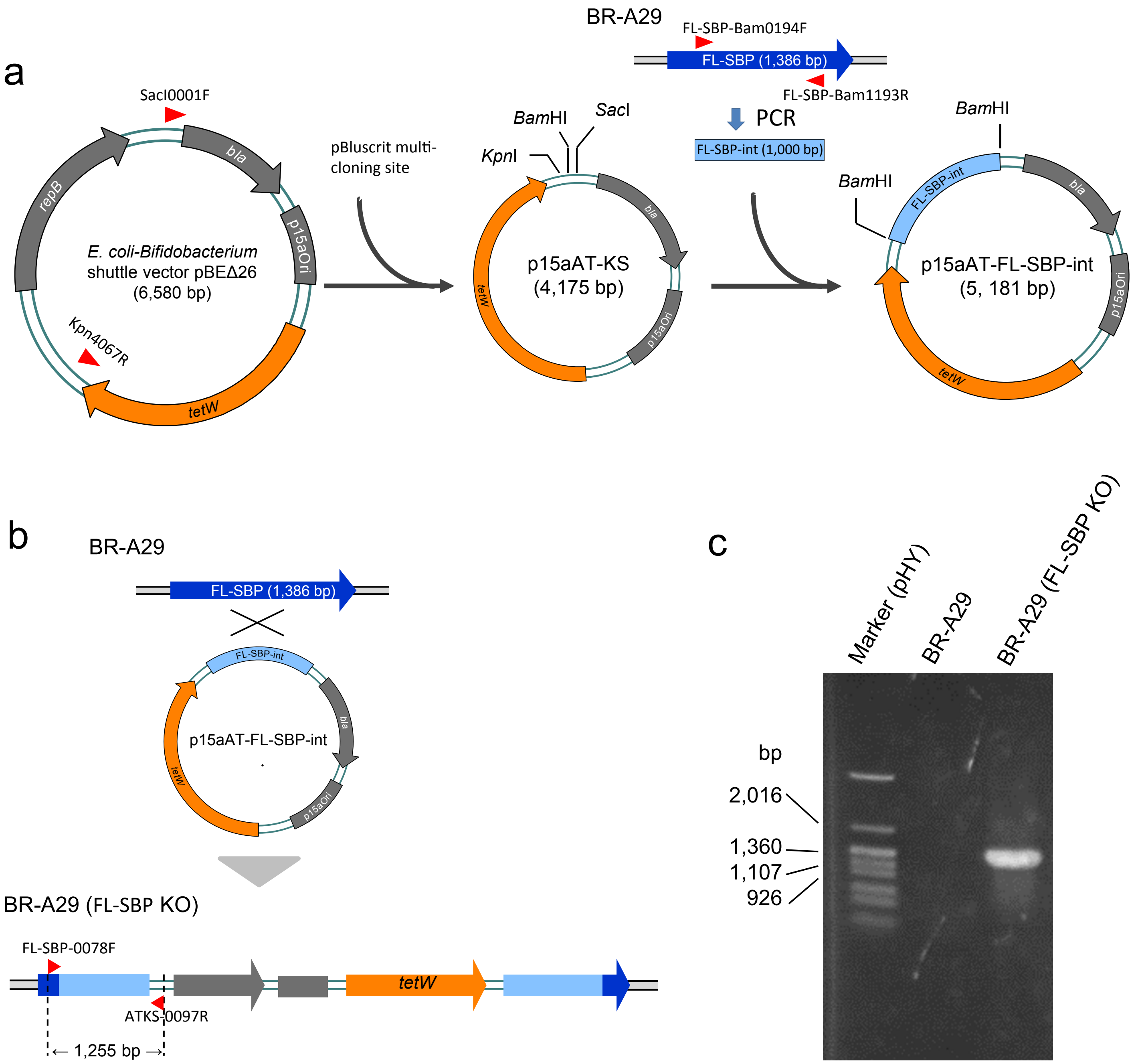

Supplementary Fig. 18. Targeted knockout of FL-SBP in *B. breve* BR-A29 by insertional mutagenesis.
a, DNA fragment was amplified from the *Escherichia coli* and *Bifidobacterium*-derived shuttle vector pBEΔ26 containing a p15A origin of replication (for *E. coli*) and the *bla* and *tetW* genes (for ampicillin and tetracycline resistance, respectively). The amplified fragment was digested with *Sac*I and *Kpn*I and ligated with multiple-cloning site of pBluescript II. An internal 1000-bp fragment of the FL-SBP gene (partial FL-SBP, denoted as 'FL-SBP-int') was inserted to construct the p15aAT-FL-SBP-int plasmid for insertional mutagenesis of the FL-SBP gene. **b,** Schematic representation of homologous recombination. **c,** The expected site-specific recombination event in a tetracycline-resistant mutant was confirmed by PCR with the FL-SBP-0078F and ATKS-0097R primers.

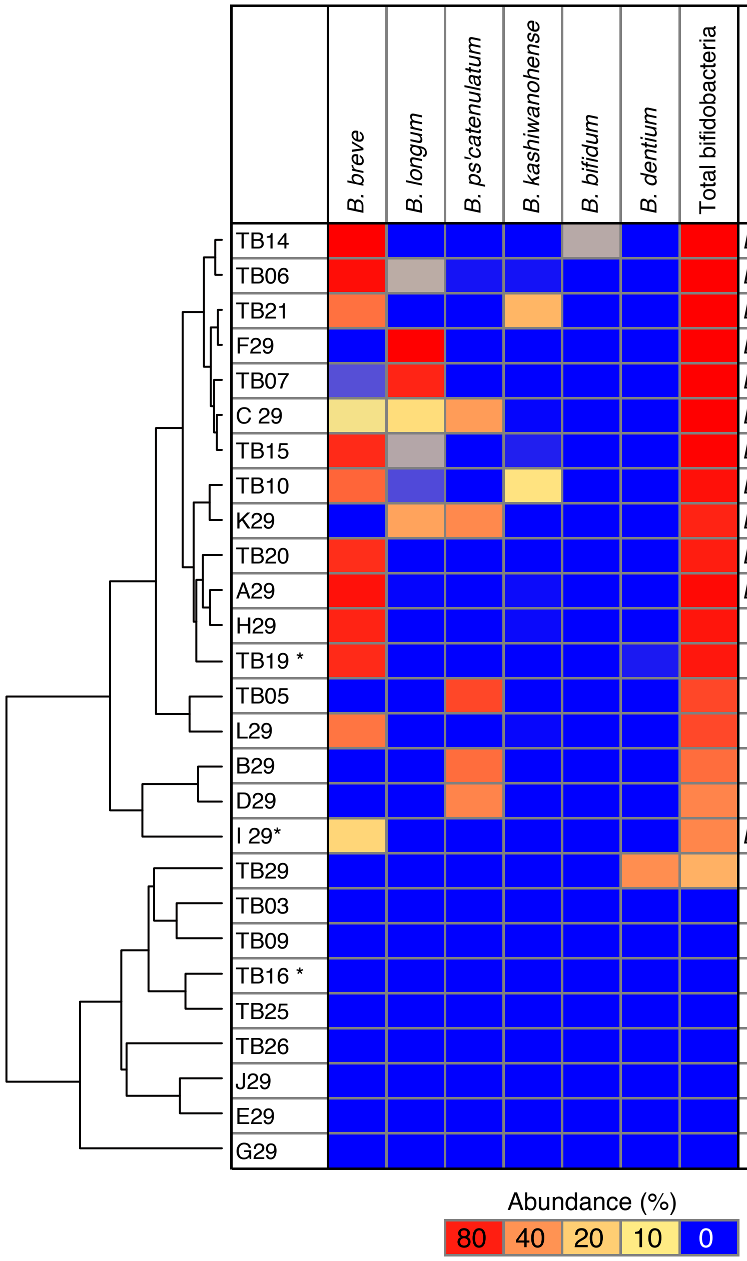

	FL utilising strains	FL non-utilising strains	Cluster
TB14	*B. breve* BR-14, *B. bifidum* BI-14		B1
TB06	*B. breve* BR-06	*B. longum* ss. *longum* LO-06	B1
TB21	*B. breve* BR-21	*B. longum ss. longum* LO-21	B1
F29	*B. longum* ss. *infantis* IN-F29	-	B1
TB07	*B. longum* ss. *infantis* IN-07	*B. breve* BR-07	B1
C 29	*B. ps'catenulatum* CA-C29	*B. breve* BR-C29, *B. longum* LO-C29	B1
TB15	*B. breve* BR-15	-	B1
TB10	*B. breve* BR-10	*B. longum* ss. *longum* LO-10	B1
K29	*B. ps'catenulatum* CA-K29a, b	*B. longum* ss. *longum* LO-K29a, b	B1
TB20	*B. breve* BR-20	-	B1
A29	*B. breve* BR-A29	-	B1
H29	-	*B. breve* BR-H29	B2
TB19 *	-	*B. breve* BR-19	B2
TB05	-	*B. ps'catenulatum* CA-05	B2
L29	-	*B. breve* BR-L29	B2
B29	-	*B. ps'catenulatum* CA-B29	B2
D29	-	*B. ps'catenulatum* CA-D29	B2
I 29*	*B. breve* BR-I29	-	B1
TB29	-	*B. dentium* DE-29	E
TB03	-	-	E
TB09	-	-	E
TB16 *	-	-	E
TB25	-	-	E
TB26	-	-	E
J29	-	-	E
E29	-	-	E
G29	-	-	E

Supplementary Fig. 19. Distribution of FL-utilising and FL-non-utilising bifidobacteria.

Bifidobacterial abundances in 1-month-old infants (n = 27) were illustrated with heatmap at species level. The clusters to which the infants belong to are also indicated. Infants marked with an asterisks received breast milk from non-secretor mothers.

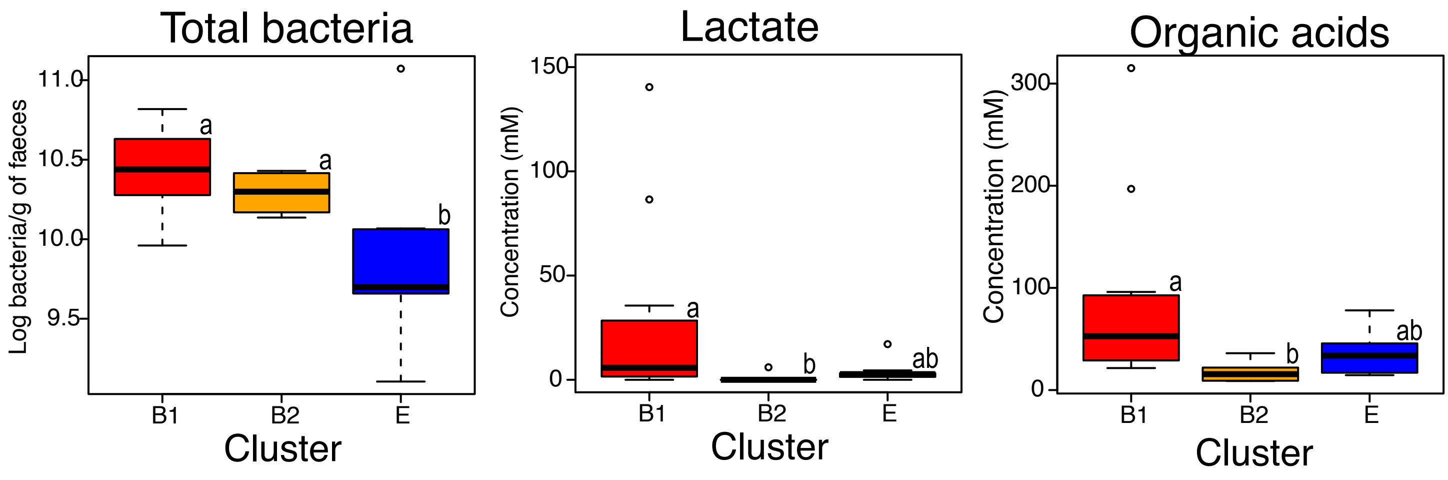

Supplementary Fig. 20. Differences in gut environments among clusters B1, B2, and E. Different letters (a–c) above the boxes indicate significant differences between clusters ($p < 0.05$, Mann–Whitney U test with Bonferroni's correction). Cluster B1, (FL-utilising Bifidobacteriaceae-predominant, red), cluster B2 (non-FL-utilising Bifidobacteriaceae-predominant, orange) and cluster E (Enterobacteriaceae-predominant, blue) were compared.

Supplementary Table 1. Relevant characteristics of infants in whom gut microbiota were investigated during the first month of life

Subject ID	Delivery	Birth weight (g)	Sex	Gestation (wk)	Sibling	Feeding (% breast feeding)		
						Formula (< 10%)	Mixed (11-89%)	Breast (> 90%)
A	Vaginal	2,820	M	40	-	days 0-3	days 4-12	days 13-30
B	Vaginal	3,030	F	40	+	days 0-2	days 3-7, 9-10	days 8, 11-30
C	Vaginal	2,620	M	39	-	days 0-1	-	days 2-30
D	Vaginal	2,750	F	38	+	day 0	days 1, 2, 4-15	days 3, 16-30
E	Vaginal	2,544	F	40	-	-	-	days 0-30
F	Vaginal	2,706	M	38	-	-	days 5-7	days 0-4, 8-30
G	Vaginal	2,525	M	38	-	-	days 13-30	days 0-12
H	Vaginal	2,935	M	38	+	-	-	days 0-30
I	Vaginal	2926	M	40	-	days 0-2	day 3	days 4-30
J	Vaginal	3178	F	38	+	day 1	days 2,3	days 4-30
K	Vaginal	3092	F	38	+	-	days 1-3	days 4-30
L	Vaginal	2960	M	39	+	-	-	days 0-30

Supplementary Table 2. Infant volunteers, samples analysed, and the number of 454 reads per sample

	Infant volunteers (n = 12)											
	A	B	C	D	E	F	G	H	I	J	K	L
Day 1	-	1,489	2,146	1,471	1,195	-	NT	NT	NT	-	NT	NT
Day 2	2,716	2,134	1,857	1,460	1,364	1,847	NT	4,070	NT	4,615	3,344	NT
Day 3	1,767	1,903	1,826	1,135	1,138	2,578	-	4,245	3,435	5,331	3,711	5,421
Day 4	1,455	1,870	1,890	2,253	3,921	1,627	NT	3,617	4,427	-	3,720	4,935
Day 5	1,981	1,564	1,660	2,015	2,605	1,319	4,406	4,793	2,577	4,210	3,780	7,476
Day 6	1,289	2,101	1,825	2,029	1,069	1,357	4,049	3,789	3,730	3,816	3,892	3,763
Day 7	1,408	2,658	2,160	1,859	1,440	1,286	4,670	3,835	3,387	4,424	3,464	3,720
Day 9	1,818	2,475	1,925	1,668	1,379	1,547	5,137	3,460	3,594	5,506	5,698	4,172
Day 11	3,059	2,953	1,801	2,474	2,252	1,309	4,185	3,820	3,796	4,126	4,217	3,690
Day 13	2,312	3,226	3,150	3,110	2,335	1,875	4,211	4,803	3,502	5,219	4,075	3,465
Day 15	2,528	2,867	2,370	1,330	2,023	1,143	2,895	4,126	2,529	4,863	4,374	8,381
Day 17	2,684	2,570	1,519	1,104	1,704	1,261	2,953	3,800	3,318	4,711	4,878	4,903
Day 19	1,990	1,817	2,517	1,293	1,432	1,430	2,009	3,876	4,479	3,734	4,602	5,344
Day 21	3,024	2,621	1,772	1,303	1,945	1,356	1,954	4,041	4,633	3,536	3,968	3,400
Day 23	2,556	2,671	1,214	1,079	1,286	1,783	2,799	4,100	5,070	4,514	4,131	5,853
Day 25	2,711	1,067	1,505	1,118	1,432	1,080	2,367	2,362	3,488	4,125	4,347	2,274
Day 27	2,982	1,594	1,610	1,483	1,467	1,248	1,898	2,330	3,580	3,897	3,642	8,124
Day 29	2,330	1,991	1,388	1,249	1,558	1,596	2,394	3,067	3,436	4,005	3,976	4,870
Total	38,610	39,571	34,135	29,433	31,545	25,642	45,927	64,134	58,981	70,632	69,819	79,791
Means	2,271	2,198	1,896	1,635	1,753	1,508	3,281	3,773	3,686	4,415	4,107	4,987

-, Sample not provided. NT, not tested since PCR product was not obtained with 40 cycle PCR

Supplementary Table 3. Analysis workflow and QIIME parameter settings used in this study

QIIME and Unix command [a]	Options
split_libraries.py [b]	-m MAP##.txt -f RunFile##.fna -q RunFile##.qual -l 200 -L350 -s 25 -a 0 -M 2 -e 1 -w 100 -b 10 -z disable --max-homopolymer 10 -o Run##/seqs.fna
truncate_reverse_primer.py [b]	-f Run##/seqs.fna -z truncate_remove -m SeqData/MAP##.txt -o Run##/ --primer_mismatches 2
cp [b]	Run##/seqs_rev_primer_truncated.fna seqs.Run##.fna
cat	*.fna > seqs.fna
pick_otus.py	-i seqs.fna -m usearch --db_filepath=/QIIME_database/gold_fa.fasta -g 2 --suppress_de_novo_chimera_detection
pick_rep_set.py	-f seqs.fna -i usearch_picked_otus/seqs_otus.txt -m most_abundant
align_seqs.py	-i seqs.fna_rep_set.fasta -m muscle
make_phylogeny.py	-i muscle_aligned/seqs.fna_rep_set_aligned.fasta -o rep_phylo.tre
assign_taxonomy.py	-i seqs.fna_rep_set.fasta -m rdp -c 0.5
make_otu_table.py	-i usearch_picked_otus/seqs_otus.txt -t rdp_assigned_taxonomy/seqs.fna_rep_set_tax_assignments.txt -o otu_table.biom
biom summarize-table	-i otu_table.biom -o reads_per_sample.txt
make_otu_heatmap_html.py	-i otu_table.biom -o otus/OTU_Heatmap/ -n 1 -m MAP.txt
summarize_taxa.py	-i otu_table.biom -o wf_taxa_summary/
plot_taxa_summary.py	-i wf_taxa_summary/otu_table_L5.txt -l Family -o graph_family -c bar --include_html_counts
multiple_rarefactions.py	-i otu_table.biom -o wf_arare/rarefaction/ --min 20 --max 5000 --num-reps 40 --step 20
alpha_diversity.py	-i wf_arare/rarefaction/ -m chao1,PD_whole_tree,observed_species,shannon -o wf_arare/alpha_div/ -t rep_phylo.tre
collate_alpha.py	-i wf_arare/alpha_div/ -o wf_arare/alpha_div_collated/
make_rarefaction_plots.py	-i wf_arare/alpha_div_collated/ -m MAP.txt -o alpha_diversity_html
single_rarefaction.py	-i otu_table.biom -o otus/otu_table_beta.biom -d 1000
make_prefs_file.py	-m MAP.txt -o prefs_out.txt

[a] MacQIIME version 1.8.0 (http://www.wernerlab.org/software/macqiime) was used in this study.

[b] These commands were used for each 454-run file.

Supplementary Table 4. Relevant characteristics of infants in whom gut microbiota were investigated at approximately 1 month after birth

ID [a]	Delivery	Birth weight (g)	Sex	Gestation (wk)	Sibling	Feeding
A29	Vaginal	2,820	M	40	-	Breast
B29	Vaginal	3,030	F	40	+	Breast
C29	Vaginal	2,620	M	39	-	Breast
D29	Vaginal	2,750	F	38	+	Breast
E29	Vaginal	2,544	F	40	-	Breast
F29	Vaginal	2,706	M	38	-	Breast
G29	Vaginal	2,525	M	38	-	Breast
H29	Vaginal	2,935	M	38	+	Breast
I29	Vaginal	2,926	M	40	-	Breast
J29	Vaginal	3,178	F	38	+	Breast
K29	Vaginal	3,092	F	38	+	Breast
L29	Vaginal	2,960	M	39	+	Breast
TB03	Vaginal	3,454	M	40	-	Breast
TB05	Vaginal	3,356	M	38	-	Breast
TB06	Vaginal	3,125	M	39	+	Breast
TB07	Vaginal	2,386	M	34	+	Breast
TB09	Vaginal	3,132	F	41	-	Breast
TB10	Vaginal	3,530	M	40	+	Breast
TB14	Vaginal	2,866	M	40	+	Breast
TB15	Vaginal	2,500	M	37	+	Breast
TB16	Vaginal	2,592	F	39	-	Breast
TB19	Vaginal	3,334	M	39	-	Breast
TB20	Vaginal	3,256	M	40	+	Breast
TB21	Vaginal	3,784	M	39	+	Breast
TB25	Vaginal	2,946	F	37	+	Breast
TB26	Vaginal	2,770	F	40	-	Breast
TB29	Vaginal	2,414	F	37	-	Breast

[a] The subject background information of 12 infants (A to L) is shown for comparison purposes here, as well as in Supplementary Table 1. “TB” is the ID of this follow-up cohort, and the numbers are sequential serial numbers. In the follow-up cohort, 30 infants were recruited, and 15 exclusively breast-fed infants were selected for the analysis.

Supplementary Table 5. Sample IDs, ages, and family relationships of the 22 adult volunteers

Infant ID	Father		Mother	
	ID	Age	ID	Age
A	faA	42	moA	41
B	faB	43	moB	42
C	faC	29	-	-
D	faD	36	moD	33
E	faE	33	moE	31
F	faF	31	moF	29
G	faG	33	-	-
H	faH	29	moH	30
I	faI	31	moI	30
J	faJ	31	moJ	31
K	faK	34	moK	34
L	faL	35	moL	33

-, sample not provided.

Supplementary Table 6. Concentration of HMO component in breast milk (mM).

Infant Subject ID	2'-FL	3-FL	DF-L	LNT	LNnT	LNFP	LNDFH I	LNDFH II
A29	5.6	0.9	0.3	2.1	0.8	2.6	1.2	0.0
B29	3.1	0.7	0.2	0.7	5.2	1.0	0.3	0.1
C29	4.5	1.0	0.4	0.8	9.5	1.3	0.8	0.4
D29	4.2	0.8	0.3	0.7	8.6	1.1	0.5	0.2
E29	5.8	1.0	0.6	0.5	8.7	1.2	0.7	0.2
F29	4.3	1.2	0.4	0.4	3.4	0.8	0.5	0.3
G29	5.6	1.1	0.5	0.4	3.5	1.2	0.5	0.5
H29	4.3	1.1	0.3	0.7	11.1	1.1	0.7	0.4
I29	0.0	2.5	0.0	1.4	3.4	1.4	0.0	0.1
J29	6.1	0.5	0.4	0.7	5.8	2.2	0.8	0.5
K29	2.9	1.7	0.4	1.1	5.1	1.4	1.1	0.2
L29	5.7	1.2	0.7	0.5	6.9	1.3	0.8	0.2
TB03	4.0	0.9	0.3	0.6	4.3	1.2	0.7	0.0
TB05	4.5	1.0	0.3	0.3	12.5	0.7	0.3	0.1
TB06	2.4	1.1	0.2	1.0	3.4	1.3	0.6	0.0
TB07	4.0	1.5	0.4	0.7	9.7	1.1	0.7	0.2
TB09	4.6	1.6	0.6	0.3	4.1	1.0	0.6	0.0
TB10	3.8	1.3	0.4	0.7	10.9	1.1	0.8	0.2
TB14	2.1	1.9	0.3	0.4	4.3	0.7	0.3	0.0
TB15	3.5	3.1	0.4	0.5	20.6	0.9	0.4	0.1
TB16	0.0	2.0	0.0	2.5	8.9	1.4	0.0	0.1
TB19	0.0	5.0	0.0	1.0	6.0	1.8	0.0	0.0
TB20	3.4	1.7	0.5	0.7	6.2	1.2	0.9	0.1
TB21	2.9	2.4	0.4	0.5	12.8	0.8	0.3	0.1
TB25	4.0	1.3	0.7	1.1	6.8	1.7	1.2	0.3
TB26	2.7	2.5	0.4	0.4	8.3	0.8	0.3	0.1
TB29	3.4	2.1	0.4	0.7	9.9	1.1	0.7	0.2

Supplementary Table 7. Summary of the draft genome of 29 bifidobacterial strains, their utilisation of fucosyllactose, the presence of genes involved in FL utilisation, and their accession numbers [a].

No.	Strains	Genome size (Mbp)	No. of CDSs	Value				Isolated from	Growth in HMO medium (OD_{600} > 0.7)	α-L-fucosidase		SBP	Accession no.
				No. Contig	Max contig (kbp):	N50[b] (kbp):	Coverage			GH29	GH95	HG_2571	
1	*B. breve* BR-06	2.7	2304	101	266	82	100x	TB06	+	-	+	+	BCXL01000000
2	*B. breve* BR-07	2.2	1955	27	471	339	70x	TB07	-	-	+	-	BCXM01000000
3	*B. breve* BR-10	2.3	2005	60	265	111	60x	TB10	+	-	+	+	BCXN01000000
4	*B. breve* BR-14	2.5	2315	57	223	117	70x	TB14	+	-	+	+	BCXO01000000
5	*B. breve* BR-15	2.4	2078	27	408	165	80x	TB15	+	-	+	+	BCXP01000000
6	*B. breve* BR-19	2.3	2012	15	575	334	100x	TB19	-	-	+	-	BCXQ01000000
7	*B. breve* BR-20	2.3	2039	52	233	115	100x	TB20	+	-	+	+	BCXR01000000
8	*B. breve* BR-21	2.6	2368	66	311	116	170x	TB21	+	-	+	+	BCXS01000000
9	*B. breve* BR-A29	2.4	1988	62	253	141	90x	A29	+	-	+	+	BCXT01000000
10	*B. breve* BR-C29	2.3	2016	41	228	147	100x	C29	-	-	+	-	BCXU01000000
11	*B. breve* BR-H29	2.5	2224	39	363	214	90x	H29	-	-	+	-	BCXV01000000
12	*B. breve* BR-I29	2.2	1928	39	196	131	80x	I29	+	-	+	+	BCXW01000000
13	*B. breve* BR-L29	2.3	2057	44	357	126	200x	L29	-	-	+	-	BCXX01000000
14	*B. dentium* DE-29	2.6	2127	26	275	191	140x	TB29	-	+	-	-	BCYE01000000
15	*B. longum* ss. *infantis* IN-07	2.7	2441	114	286	86	310x	TB07	+	+	+	+	BCYF01000000
16	*B. longum* ss. *infantis* IN-F29	2.6	2356	58	241	131	90x	F29	+	+	+	+	BCYG01000000
17	*B. longum* ss. *longum* LO-06	2.4	2034	77	329	72	40x	TB06	-	-	-	-	BCYH01000000
18	*B. longum* ss. *longum* LO-10	2.5	2153	80	275	113	100x	TB10	-	-	-	-	BCYI01000000
19	*B. longum* ss. *longum* LO-21	2.7	2209	71	480	131	160x	TB21	-	-	-	-	BCYJ01000000
20	*B. longum* ss. *longum* LO-C29	2.5	2073	49	224	112	110x	C29	-	-	-	-	BCYK01000000
21	*B. longum* ss. *longum* LO-K29a	2.4	2030	85	268	93	160x	K29	-	-	-	-	BCYL01000000
22	*B. longum* ss. *longum* LO-K29b	2.4	1987	97	283	92	300x	K29	-	-	-	-	BCYM01000000
23	*B. pseudocatenulatum* CA-05	2.2	1896	24	449	272	140x	TB05	-	-	-	-	BCXY01000000
24	*B. pseudocatenulatum* CA-B29	2.3	1904	43	440	354	160x	B29	-	-	-	-	BCXZ01000000
25	*B. pseudocatenulatum* CA-C29	2.2	1825	72	312	115	60x	C29	+	-	+	+	BCYA01000000
26	*B. pseudocatenulatum* CA-D29	2.3	1903	86	292	84	60x	D29	-	-	-	-	BCYB01000000
27	*B. pseudocatenulatum* CA-K29a	2.5	2168	59	576	249	230x	K29	+	-	+	+	BCYC01000000
28	*B. pseudocatenulatum* CA-K29b	2.5	2148	66	367	122	70x	K29	+	-	+	+	BCYD01000000
29	*B. bifidum* BI-14	2.2	1779	26	263	157	120x	TB14	+	+[c]	+[c]	-	BCXK01000000

[a] These draft genome sequences were deposited in the DDBJ WGS database under BioProject Accession No. PRJDB4597.
[b] N50; the length of the smallest contig within the set of longer contigs, which represent at least half of the cumulative lengths of all contigs.
[c] Only the fucosidase from *B. bifidum* BI-14 is predicted to be an extracellular membrane protein.
